# Safety and Efficacy of Gammaplex® in Idiopathic Thrombocytopenic Purpura (ClinicalTrials.gov - NCT00504075)

**DOI:** 10.1371/journal.pone.0096600

**Published:** 2014-06-03

**Authors:** Clive H. Dash, Kate R. Gillanders, Margaret E. Stratford Bobbitt, Ernie W. Gascoigne, Samantha J. Leach

**Affiliations:** Medical Department, Bio Products Laboratory Limited (Ltd.), Elstree, Hertfordshire, United Kingdom; National Cancer Institute, United States of America

## Abstract

**Background and Objectives:**

This multicentre, open-label study investigated the safety and efficacy of Gammaplex, a 5% Intravenous Immunoglobulin (IVIg), in patients with idiopathic (immune) thrombocytopenic purpura (ITP).

**Materials and Methods:**

Patients were between the ages of 6 and 70 years; had ITP for at least six months and had a platelet count ≤20×10^9^/L. Eligible patients were dosed with 1 g/kg of Gammaplex on two consecutive days, followed by assessment of safety and efficacy on Days 3, 5, 9, 14, 21, 32 and 90. Response was defined as the increase in platelet count to a threshold of ≥50×10^9^/L on or before Day 9 after the first dose of Gammaplex.

**Results:**

All 35 patients received at least one infusion of Gammaplex. Twenty-nine (83%) patients responded to Gammaplex, similar to the historical control, with a 95% lower one-sided confidence interval of 68.9%. Median duration of response was 10.0 days, with an overall reduction in bleeding episodes. Gammaplex provided supranormal concentrations of total IgG; mean peak concentration (Cmax) of 45.3 g/L (4.53 g/dL), with a mean half-life of 28.5 days. Fifteen patients reported 63 Adverse Drug Reactions (ADRs); the most common were headache (10 patients), vomiting (6 patients) and pyrexia (5 patients). Five of these ADRs were considered serious, one patient had three concurrent Serious Adverse Events (SAEs); these were vomiting, dehydration and headache. Two other patients each had one SAE (headache). There were no unexpected Adverse Events (AEs) or thromboembolic episodes and no significant changes in vital signs, biochemical, haematological and virology results. Conclusion: Gammaplex achieved a very high concentration of serum IgG but was well-tolerated and effective in the treatment of ITP with a similar degree of efficacy to the pre-determined historical control group and the pre-set statistical criteria.

**Trial Registration:**

ClinicalTrials.gov NCT00504075 Clinical Trials Registry India 000016

## Introduction

Idiopathic (immune) thrombocytopenic purpura (ITP) is an autoimmune disorder affecting both children and adults; it is characterised by a low platelet count, normal results on a bone marrow examination (except possibly for increased megakaryocytes) and the absence of specific causes of thrombocytopenia, such as leukaemia, aplastic anaemia or disseminated intravascular coagulation [1]–[3]. Childhood ITP is typically of acute onset. In more than 70% of children, spontaneous and permanent remission occurs within one year of onset [3], [4]. In contrast, the majority of adults have persistent ITP, although the natural history is less defined than that for ITP in children, and some patients do improve with time [5], [6]. Rarely, life-threatening bleeding occurs, but when it does, intracranial haemorrhage is the principal cause of death [7]. It is generally recognised that serious haemorrhage is most likely to occur when the platelet count falls below 20×10^9^/L [2], [3], [6]. Other bleeding may occur and may result in acute or chronic anaemia. The use of IVIg to increase the platelet count rapidly has been shown in other studies to decrease signs and symptoms of haemorrhage. The first report of the efficacy of IVIg in the treatment of ITP appeared in 1981 [8]. More than 100 studies have subsequently confirmed the safety and efficacy of IVIg as treatment of ITP in children and adults [9]–[14]. A substantial and rapid platelet increase can be achieved with an IVIg dosage of 1 g/kg per day repeated for two consecutive days, and this is now the preferred regimen [11], [15], [16].

Gammaplex, a highly purified, unmodified IVIg, manufactured by BPL from human plasma which is processed by cold ethanol fractionation and chromatography. The process also includes three viral inactivation/removal steps, namely solvent/detergent treatment, nanofiltration (20 nm) and a terminal low pH incubation of the finished product to enhance safety [17].

The purpose of this study was to investigate the efficacy and safety of Gammaplex in patients with ITP. Response to Gammaplex treatment was assessed by the increase in platelet count to a threshold of ≥50×10^9^/L and the duration of the response compared to historical controls [5], [7]–[18]. In addition, pharmacokinetic data are presented for Gammaplex after this high dosage in patients with ITP. Provan et al [19] and Rodeghiero et al [20] reported changes to the definition of types of ITP, following some international reviews. This manuscript briefly discusses these reviews and how this study (GMX02) complies with the revised guidelines.

The product used in this study was a ready-prepared solution for intravenous (IV) administration that contained 5 g human normal immunoglobulin and 5 g D-sorbitol (as a stabiliser) in 100 mL of buffer solution containing: 0.6 g glycine, 0.2 g sodium acetate, 0.3 g sodium chloride and approximately 5 mg Polysorbate 80.

## Materials and Methods

The protocol for this trial and supporting TREND checklist are available as supporting information; see Checklist S1 and Protocol S1.

### Ethical Statement

This study (GMX02) was conducted in accordance with the ethical principles that have their origins in the Declaration of Helsinki. The study was conducted in compliance with the protocol, International Conference on Harmonisation (ICH) harmonised tripartite guideline E6(R1): Good Clinical Practice regulations and all applicable regulatory requirements.

The study protocol, patient information, consent form and any other materials provided to the patients were submitted by the investigators to their institutional review board (IRB) or independent ethics committee (IEC). Written approval from the IRB or IEC was available before any patient was enrolled.

Written informed consent was obtained from all patients by the site staff prior to enrolment into the study.

### Regulatory and Ethical Approvals

This study (GMX02) was reviewed and approved by the US Department of Health and Human Services, Center for Biologics Evaluation and Research (Food and Drug Administration), Department of Health for Argentina, Argentine National Food, Drug and Medical Technology Administration and Directorate General of Health Services, Office of Drugs Controller General (India).

For all the recruiting centres ethical approval for the study was obtained from the following Ethics Committees (ECs)/IRBs:

Schulman Associates IRB, Inc., Central IRB, USA (Dr Lyons, Dr Ortega, Dr Priego); Oregon Health and Science University, Portland, USA (Dr Boshkov); Chesapeake Research Review, Inc., Columbia, USA (Prof Parker); CER San Juan, Center for Clinical Research and Assistance, Teaching and Research Committee, San Juan, Argentina (Dr Gomez); Research EC of Hospital Italiano de la Plata, La Plata, Argentina (Dr Enrico); Department of Public Health, Research Department at Hospital Dr José Ramón Vidal, Corrientes, Argentina (Dr Lanari); Mallikatta EC, Mangalore, India (Dr Alva); Institutional EC, Kasturba Medical College, Mangalore, India (Dr Chakrapani); EC of Manipal Hospital & Manipal Heart Foundation, Bangalore, India (Dr Dixit); Human Research EC, M.S. Patel Centre for Medical Care and Education, Karamsad, India (Prof Haritha); EC of Mahavir Hospital and Research Centre, Hyderabad, India (Dr Kulkarni); Institutional EC, Deenanath Mangeshkar Hospital and Research Centre, Pune, India (Dr Melinkeri); Ethical Review Board, M.S. Ramaiah Medical College and Teaching Hospital, Bangalore, India (Dr Narayan); EC, Apollo Health City, Hyderabad, India (Dr Prasad), EC of The Heart Care Clinic, Ahmedabad, India (Dr Shah); Siddhant IEC, Ahmedabad, India (Dr Sharma).

### Study design

This was a multicentre, open-label, non-randomised, prospective study. Sample size was calculated by using the normal approximation for a single proportion within the nQuery Adviser Sample Size software (Statistical Solutions, Cork, Ireland). As per the Bussel et al study the threshold for response was a platelet count of ≥50×10^9^/L on or before Day 9 and an expected response rate of 83% based on the historical control study [11]. For a power of 80%, a two-sided type-1 error of 0.05 and a lower bound of a one-sided 95% CI (confidence interval) of at least a 60% response rate, using the normal approximation to the binomial distribution, a minimum of 31 patients needed to be studied.

In the final analysis, the exact method was used to calculate the response rate including the lower one-sided 95% CI since the objective was to evaluate non-inferiority versus the historical control.

All the patients were screened and dosed at the study sites. After screening and enrolment, patients received infusions of Gammaplex on Day 1 and Day 2 (first treatment course). Efficacy assessments (platelet counts) were conducted prior to treatment and on Days 3, 5, 9, 14, 21 and 32 and a final follow-up visit on Day 90 (End of Study). Safety assessments, including vital signs and laboratory tests, were performed at the same time points. During the study, patients had to complete diary cards daily between Day 1 and Day 32, mentioning any AEs, concomitant medications, and the presence/absence of bleeding episodes.

### Patient selection

Patients were initially approached by an investigator. Enrolment into the study commenced in September 2007 and last patient last visit was conducted in August 2011. Patients were eligible if they: were between the ages of six and 70 years; had ITP for at least six months; had a platelet count ≤20×10^9^/L just before the first infusion of Gammaplex; no other conditions that could cause thrombocytopenia and no history of intolerance to blood, blood-derived products or any other IgG (immunoglobulin) preparation; were not pregnant or nursing.

Exclusion criteria included: had received any live virus vaccine or rituximab within the previous three months; received an IVIg preparation or investigational agent or any blood, blood product, or blood derivative within one month before Day 1; had any history or signs of hyperviscosity, transient ischaemic attack, stroke, other thromboembolic event or unstable angina, deep vein thrombosis; had abused drugs within the previous 12 months, had uncontrolled arterial hypertension, anaemia, severe renal or liver impairment; tested positive for Hepatitis B, C or HIV; suffered from any acute or chronic medical conditions (e.g. renal disease or predisposing conditions for renal disease, coronary artery disease or protein losing enteropathy).

Patients on corticosteroids were eligible for the study, however they had to be on stable therapy for at least two week prior to dosing with Gammaplex and any changes to therapy were to be avoided during the study.

There were some protocol deviations noted, the majority were related to procedures, such as vital signs or laboratory samples not being conducted/processed correctly (47 incidences), the second highest were related to visit schedules being outside the visit windows set (18 incidences). There were 10 incidences in 5 patients relating to errors in dosing calculations or infusion timing which resulted in less than 1 g/kg of Gammaplex being administered. Also, three incidences related to wrong filter sizes being used. Two other protocol deviations were related to enrolment criteria. Two separate incidences of inadequate consenting were noted. One additional patient was reported to have had prohibited medication. All the deviations were logged and appropriate follow-up action was taken.

### Treatment

Each patient was scheduled to receive 1 g/kg of Gammaplex on each of two consecutive days per course; this was in accordance with the Committee for Medicinal Products for Human use (CHMP) Note for Guidance recommendations on dosage regimen for ITP [21]. If the platelet count was not maintained for the desired length of time after the first course, subjects were permitted to receive a second treatment course (Gammaplex 1 g/kg on two consecutive days) within 32 after the first dose, and then a further third course (Gammaplex 1 g/kg on two consecutive days) up to 90 days after the first dose if there was a relapse of platelet counts.

All infusions started at a rate of 0.01 mL/kg per minute for the first 15 minutes; if tolerated, the rate of infusion was increased every 15 minutes to a maximum rate of 0.08 mL/kg per minute. Lack of tolerance was recorded as an AE at the rate it first occurred.

### Analysis of Data

The intent-to-treat (ITT) and safety populations included all 35 patients who received at least one infusion of Gammaplex.

The primary efficacy variable was the response rate, *i.e.* the percentage of patients attaining a platelet count of ≥50×10^9^/L by Day 9 (the seventh day after completing the second infusion); efficacy was only assessed on the first course of Gammaplex but safety after all courses.

Secondary efficacy variables were duration of time for which the platelet count remained ≥50×10^9^/L and changes in any bleeding/haemorrhage up to Day 32. The duration of response was analysed using the Kaplan-Meier estimate of the probability of response continuing to time (t) as well as the 95% CI for the median [22]. Descriptive statistics were used to display platelet count at Day 1 (baseline), Days 2 (pre-infusion), 3, 5, 9, 14, 21 and 32.

### Pharmacokinetic analysis (PK)

Blood samples were collected during the follow-up days to measure total serum IgG. A PK analysis of baseline-adjusted serum IgG data was conducted, using a non-compartmental model (WinNonLin, NCA Model 201, Pharsight Corporation, USA). Overall descriptive statistics of PK parameters excluded patients with insufficient PK samples and those patients for whom the R-squared (adjusted) value was <0.8.

Safety variables included AEs, vital signs and clinical laboratory tests.

## Results

### Patients

**Figure 1:**
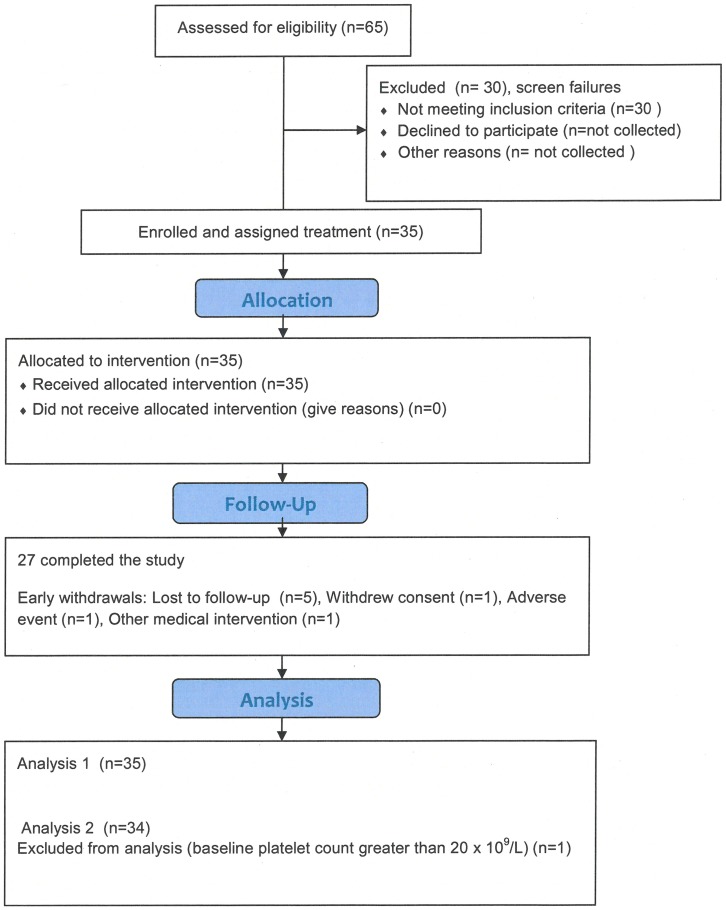
Flow diagram for patients enrolled.

A total of 65 patients were screened for the study, 30 of which did not meet the inclusion/exclusion criteria. Therefore 35 patients were enrolled in the study (Figure 1). The median age was 32 years: two were 6–11 years of age, one 12–17 years and the remainder aged 18 to 69 years (Table 1).

**Table 1:** Patients demographics.

Demographic Characteristic	N = 35
	n (%)
Age (years)	
Mean (SD)	36.3 (18.34)
Median	32.0
Min, Max	6, 69
Age group, n (%)	
6–11 years	2 (5.7)
12–17 years	1 (2.9)
18–70 years	32 (91.4)
Sex, n (%)	
Male	9 (25.7)
Female	26 (74.3)
Ethnicity^a^, n (%)	
African-American	0
Asian	1 (2.9)
Caucasian	5 (14.3)
Hispanic	3 (8.6)
Ethnicity^b^, n (%)	
Asian: Hispanic or Latino	0
Asian: Non-Hispanic or Non-Latino	21 (60.0)
Caucasian: Hispanic or Latino	5 (14.3)
Caucasian: Non-Hispanic or Non-Latino	0
Height (cm)	
Mean (SD)	156.17 (13.598)
Median	156.00
Min, Max	117.4, 181.5
Weight (kg)	
Mean (SD)	64.58 (21.609)
Median	65.00
Min, Max	17.4, 133.0

Max = maximum; Min = minimum; SD = standard deviation.

a Case report form version 1.0 captured ethnicity only.

b Case report form version 2.0 captured both race and ethnicity.

Patients were predominantly female (26 patients, 74.3%). Twenty-one patients were Asian (non-Hispanic/non-Latino) and the remainder Caucasian or Hispanic.

Median time since diagnosis of ITP was 27.8 months (range of 6 to 567 months). At screening, 26 of the 35 (74%) patients enrolled had been diagnosed with ITP for more than a year. The median platelet count just before the first infusion of Gammaplex (Day 1) was 15.0×10^9^/L (range 2 to 22×10^9^/L).

Seven patients (20.0%) had had a splenectomy. None of the patients had a history of leukemia or aplastic anemia.

### Dose of Gammaplex

All 35 patients received at least one infusion of Gammaplex, and all except one patient completed the first course of treatment. Eleven patients (31.4%) began a second course, and two patients (5.7%) began a third course. Overall, 48 treatment courses were administered.

Doses ranged from 481.5 to 1149.4 mg/kg per infusion or from 20 g to 134 g.

### Response to Gammaplex therapy

Twenty-nine of 35 patients (82.9%) responded to Gammaplex; the lower 95% CI was 68.9%. The response rate was noted to be greater in patients without a history of splenectomy (25 of 28 patients, 89.3%) than those who had undergone a splenectomy (four of seven patients, 57.1%), but the difference was not statistically significant (p = 0.079).

One subject had a platelet count just before the first infusion of 22×10^9^/L and had evidence of bleeding at the time of enrolment, so was considered ineligible according to the protocol (Figure 1). If this subject is excluded from the analysis of response rate, the mean rate is 82.4% (lower 95% CI is 68.1%), so above the minimum pre-set in the study.

**Figure 2:**
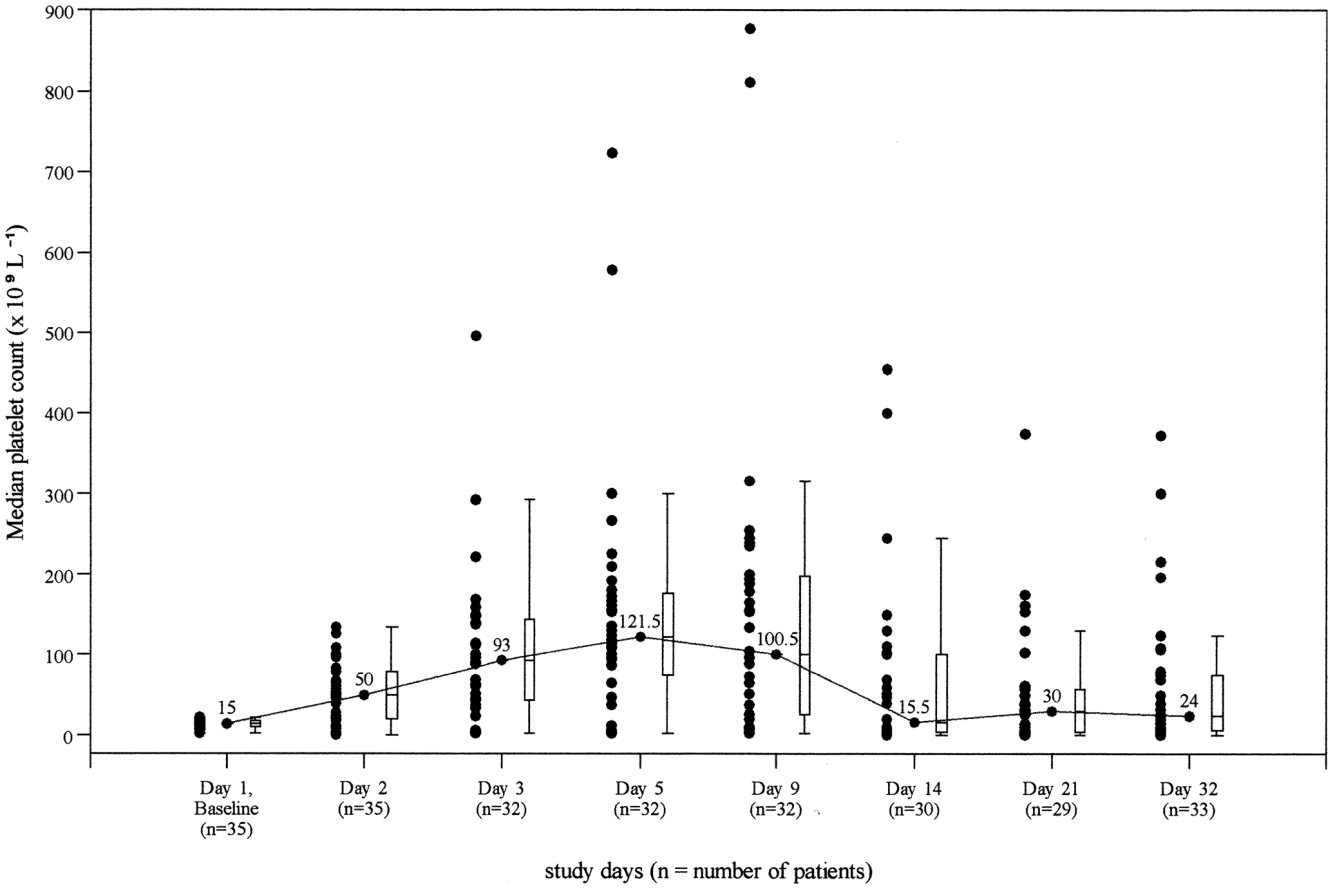
Median platelet count from baseline to Day 32 after Gammaplex infusions.

The mean (Standard Deviation, SD) platelet count value increased from 13.2 (5.95)×10^9^/L at baseline to 52.4 (36.43)×10^9^/L before infusion on Day 2 and up to 158.3 (201.27)×10^9^/L on Day 9 before decreasing to 70.2 (112.95)×10^9^/L on Day 14 and 55.2 (80.03)×10^9^/L on Day 21. The mean (SD) platelet count on Day 32 was 61.1 (89.84)×10^9^/L, which was 47.8 (89.23)×10^9^/L greater than at baseline. Figure 2 displays the median platelet count.

Eleven of 33 patients (33.3%) continued to show a response with platelet counts 50×10^9^/L or above on Day 32. Mean platelet count values on Day 32 remained higher than at baseline. The median duration of response with Gammaplex was 10.0 days.

### Pharmacokinetic (PK) analysis

Data from 25 out of the 35 patients were included into the PK analysis: 4 patients had insufficient samples; data from 6 other patients had an R-square value of <0.8. The mean peak IgG concentration (Cmax) was 4.53 g/dL (45.3 g/L), with a range of 3.11 g/dL to 7.87 g/dL. The mean half-life was 28.5 days (median 23.0 days; range 16.5 to 64.8 days); the difference between the mean and median suggests a non-normal distribution and the same applies to mean residence time (MRT), see Table 2. For clearance and volume of distribution (Vz), the mean and median values are close together, so a normal distribution may be a reasonable assumption.

**Table 2:** Gammplex pharmacokinetic data in ITP patients.

PK variable	Patient (n)	Statistic					
		Mean	SD	Median	Min	Max	95% CI
**Cmax** (g/dL)	25	4.53	1.169	4.25	3.11	7.87	4.12, 5.02
**Tmax** (Days)	25	3.12	0.440	3	3	5	2.94, 3.38
**AUC_1–29_** (days.g/dL)	25	70.3	9.14	69.0	55.8	90.0	65.45, 73.60
**Half-life** (Days)	25	28.5	12.31	23.0	16.5	64.8	25.27, 35.84
**Clearance** (mL/day/kg)	25	1.53	0.403	1.55	0.79	2.15	1.29, 1.61
**Vz** (dL/kg)	25	0.57	0.102	0.58	0.37	0.78	0.53, 0.62
**K elim** (per day)	25	0.028	0.0087	0.030	0.011	0.042	0.02, 0.03
**MRT_0-inf_** (Days)	25	40.8	16.74	34.3	25.2	91.5	36.45, 50.88

Cmax = peak concentration, Tmax = time of peak concentration, AUC = area under the curve, Vz = volume of distribution, K elim = elimination rate constant, MRT = mean residence time, CI = confidence interval.

### Bleeding/haemorrhage episodes

**Figure 3:**
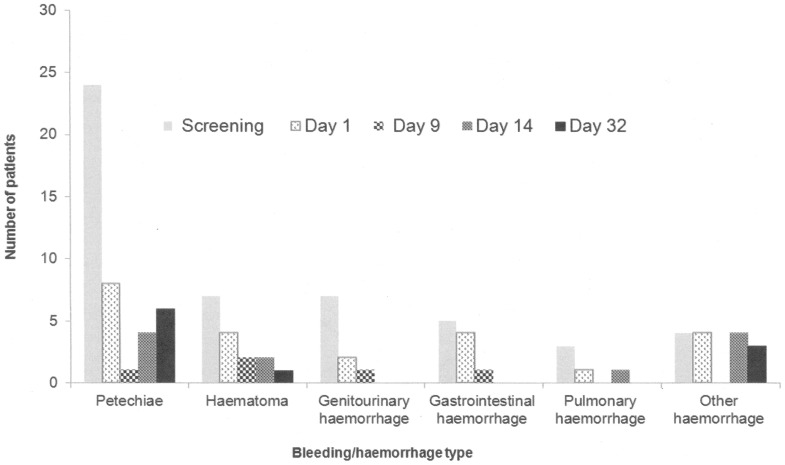
Bleeding/haemorrhage reports from screening to Day 32.

The overall incidence of bleeding or haemorrhage decreased after treatment with Gammaplex (Figure 3). At Screening (when patients had a platelet count of ≤20×10^9^/L), bleeding events reported by 10% or more patients were petechiae (24 patients, 68.6%), haematoma (7 patients, 20.0%), genitourinary haemorrhage (7 patients, 20.0%), gastrointestinal haemorrhage (5 patients, 14.3%) and other haemorrhage (4 patients, 11.4%). By Day 9, no haemorrhagic events were reported by more than 3 patients (10%) or more patients. Two Grade 3 (according to Common Terminology Criteria for Adverse Events, CTCAE) [23] bleeding events were reported between Days 2 and 32: genitourinary haemorrhage and other haemorrhage were reported by one subject (3.4%) each. Grade 2 bleeding events reported included petechiae (7 patients, 20.0%), other haemorrhage (4 patients, 11.4%), haematoma, genitourinary haemorrhage and pulmonary haemorrhage (2 patients each, 5.7%). Grade 1 bleeding events reported included haematoma (6 patients, 17.1%), petechiae (5 patients, 14.3%), other haemorrhage (4 patients, 11.4%) and pulmonary haemorrhage (one patient, 2.9%).

### Safety and tolerability

Fifteen patients (42.9%) reported 63 ADRs. The most common ADRs were headache (10 patients), vomiting (6 patients) and pyrexia (5 patients). Five of these ADRs were considered serious; one patient had three concurrent SAEs (vomiting, dehydration and headache) and two patients each had one SAE (headache). One of these latter two patients discontinued from the study. Table 3 displays ADRs reported more than once.

**Table 3:** List of Adverse Drug Reactions (ADRs) reported on more than one occasion.

Total number of patients = 35		
MedDRA Preferred Term	Number of patients (%)	Number of Events
Headache	10 (28.6)	19
Vomiting	6 (17.1)	8
Pyrexia	5 (14.3)	6
Palpitations	1 (2.9)	3
Nausea	3 (8.6)	3
Arthralgia	2 (5.7)	3
Diarrhoea	1 (2.9)	2
Dehydration	2 (5.7)	2

MedDRA = Medical Dictionary for Regulatory Activities.

The number of patients with AEs (regardless of causality) remained relatively stable at infusion rates of 0.01 mL/kg per minute and up to 0.06 mL/kg per minute, with no more than two patients experiencing AEs at any given infusion at a rate of up to 0.06 mL/kg per minute. At the maximum rate of 0.08 mL/kg per minute, the number of patients with AEs during any infusion increased to 19 out of 35 patients (54.3%).

Overall, there were no marked changes in vital signs which might indicate a significant physiological stress for any of the patients.

## Discussion

The historical comparison for the primary end-point (response rate) for this study was a large study comparing Gamunex with Gamimune N by Bussel et al [11], which had been published when this study was being designed, and had a similar dosage regimen to this study. In the above study [11], the response rate (a platelet count of ≥50×10^9^/L on or before Day 9) of the established product, Gamimune N, was 83%. Gammaplex had the same response rate 82.9% (29/35 patients) and achieved the pre-determined statistical outcome.

The duration of response was analysed slightly differently in this study compared with Bussel et al [11]. In their study, the duration of response was calculated as the proportion of patients who retained an elevated platelet count (≥50×10^9^/L) for at least seven days, and this was achieved by 60% of patients. In this study, we continued to measure platelet counts to Day 32 when the mean platelet count (61.1×10^9^/L) was still higher than the baseline value, and 11 of 33 patients (33.3%) continued to show a response with platelet counts of 50×10^9^/L or greater.

In the study used as the historical control [11], 59% of the patients had chronic ITP; the others had acute ITP. The authors reported that there was no difference in responses between these two sub-populations. Patients with acute ITP were not eligible for our Gammaplex study because of the uncertainty of whether any increase in platelet count might be related to the medication or a result of a spontaneous resolution of the condition. The latter is more common in children with ITP, and in the study by Bussel et al [11], 24% of study participants were aged less than 18 years at entry to the study. It is uncertain how many of their patients would meet the new definition of chronic ITP [20].

Bussel et al (2004) analysed bleeding as ecchymoses or petechiae over a 23 day period; 47% and 31% of patients, respectively, experienced these events [11]. In this Gammaplex study, a wider range of bleeding manifestations were monitored and showed a continuing reduction over a 32 day period. Although these data cannot be directly compared with those of Bussel et al [11], they do confirm a clinical improvement in the patients after receiving Gammaplex. In general, the infusions were well-tolerated but there was a suggestion of more ADRs at the highest rate. The highest rate of infusion was given for a substantially longer period of time than the other rates, so it is not possible to determine whether it was the rate or duration of infusion which had the major effect on ADR incidence. Out of the 94 infusions administered, 90 (96%) infusions reached and completed at the maximum recommended rate.

The PK characteristics of Gammaplex in patients with ITP were also examined. The mean and median half-life of Gammplex in this study were 28.5 and 23.0 days respectively. The overall conclusion is that Gammaplex exhibits the PK characteristics expected for an IVIg product [18], even when large doses are administered in the treatment of ITP.

There have been some changes suggested for the definition of types of ITP [19], [20]. ITP is now suggested to be categorised as chronic if it has been present for at least 12 months, which would have been the case for 26 (74%) of our 35 patients. Response has also been redefined and extended such that “a response” is now a doubling of the platelet count, although a new, less stringent entry criterion of a baseline of ≤30×10^9^/L has been recommended for clinical trials [20]. In addition, a new concept of complete response (platelets >100×10^9^/L without bleeding) has been introduced. In this study (GMX02), a doubling of platelet count was achieved by 33 (94%) patients and a value of 100×10^9^/L or more by 26 (74%) patients. The new recommendations are more onerous for clinical trials as more frequent measurements of platelet counts are needed than in this Gammaplex study to provide consistency of platelet counts before categorising responses; to comply will require daily platelet counts during at least some of the post-treatment follow-up. Platelet counts were not assessed as frequently in the Gammaplex study nor, indeed, in any prior studies of IVIg.

From the limited comparison which is possible using the new criteria in the more recent guidelines [19], [20], the overall outcome is similar but there was no suitable historical control study to fully test this conclusion.

In conclusion, Gammaplex was shown to be an effective treatment for patients with ITP with a similar degree of efficacy to the pre-determined historical control group and the pre-set statistical criteria.

## Supporting Information

Checklist S1 **CONSORT Checklist.**
(PDF)

Protocol S1 **Trial Protocol.**
(PDF)
