# Supplementary material for: Safety and Efficacy of Gammaplex® in Idiopathic Thrombocytopenic Purpura (ClinicalTrials.gov - NCT00504075)
Source: PLoS One. 2014 Jun 3;9(6):e96600. doi: 10.1371/journal.pone.0096600 (PMC4043496; doi:10.1371/journal.pone.0096600)
Supplement: Checklist S1 — CONSORT Checklist. (PDF) [file pone.0096600.s001.pdf]

Safety and Efficacy of Gammaplex in Idiopathic Thrombocytopenic Purpura (ClinicalTrials.gov – NCT00504075, Clinical Trials Registry India CTRI/2010/091/000016)

TREND Statement Checklist

Paper Section/ Topic	Item No	Descriptor	Reported?	
			✓	section
**Title and Abstract**				
Title and Abstract	1	• Information on how units were allocated to interventions	✓	Abstract
		• Structured abstract recommended	✓	Abstract
		• Information on target population or study sample	✓	Abstract
**Introduction**				
Background	2	• Scientific background and explanation of rationale	✓	Introduction
		• Theories used in designing behavioral interventions		not applicable
**Methods**				
Participants	3	• Eligubility criteria for participatants, including criteria at different levels in recruitment/sampling plan (e.g., cities, clinics, subjects)	✓	Materials and Methods: patient selection)
		• Method of recruitment (e.g., referral, self-selection), including the sampling method	✓	Materials and Methods: patient selection
		• Recruitment setting	✓	Materials and Methods: regulatory and ethics approval; patient selection
		• Settings and locations where the data were collected	✓	Materials and Methods: regulatory and ethics approval
Interventions	4	• Details of the interventions intended for each study condition and how and when they were actually administetered, specifically including:	✓	Materials and Methods: treatment
		◦ Content: What was given?	✓	Materials and Methods: treatment
		◦ Delivery method: how were the subjects grouped during delivery?	✓	Materials and Methods: treatment
		◦ Deliverer: who delivered the intervention?	✓	Materials and Methods: patient selection
		◦ Unit of delivery: how were the subjects grouped during delivery?	✓	Materials and Methods: patient selection
		◦ Setting: where was the intervention delivered?	✓	Materials and Methods: patient selection
		◦ Exposure quantity and duration: how many sessions or episodes or events were intended to be delivered? How long were they intended to last?	✓	Materials and Methods: study design
		◦ Time span: how long was it intended to take to deliver the intervention to each unit?	✓	Materials and Methods, treatment and study design
		◦ Activities to increase compliance or adherence (e.g., incentives)		none
Objectives	5	• Specific objectives and hypotheses	✓	Introduction
Outcomes	6	• Clearly defined primary and secondary outcomes measures	✓	Materials and Methods: data analysis, and Results
		• Methods used to collect data and any methods used to enhance the quality of measures	✓	Materials and Methods: study design, and Results
		• Information on validated instruments such as psychometric and biometric properties		not applicable
Sample Size	7	• How sample size was determined and,when applicable, explanation of any interim analyses and stopping rules	✓	Materials and Methods: study design
Assignment Method	8	• Unit assigment (the unit being assigned to study condictions, e.g., indvidual, group, community)	✓	Results: patients; doses of gammaplex
		• Method used to assign units to study conditions, including details of any restrictions (e.g., blocking, stratification, minimization)		not applicable
		• Inclusion of aspects employed to help minimize potential bias induced due to non-ramdomized (e.g., matching)	✓	Materials and Methods: patient selection

TREND Statement Checklist				
Blinding (masking)	9	• Whether or not participants, those administering the intervention, and those assessing the outcomes were blinded to study condictions assignment; if so, statement regarding how the blinding was accomplished and how it was assessed.		not applicable
Unit of Analysis	10	• Description of the smallest unit that is being analyzed to assess intervention effects (e.g., individual, group, or community)	✓	Materials and methods: data analysis
		• If the unit of analyses differs from the unit of assigment, the analytical method used to account for this (e.g., adjusting the standard error estimates by the design effect or using multilevel analysis)		not applicable
Statistical Methods	11	• Statistical methods used to compare study groups for primary methods outcomes(s), including complex methods of correlated data	✓	Materials and methods: data analysis
		• Statistical methods used for additional analysis, such as a subgroup analyses and adjusted analysis	✓	Results: pharmacokinetics analysis
		• Methods for imputing missing data, if used		N/A
		• Statistical software or programs used	✓	Materials and Methods: study design; data analysis
**Results**				
Participant flow	12	• Flow of participants through each stage of the study: enrollment, assignment, allocation, and intevention exposure, follow-up, analysis (a diagram is strongly recommended)	✓	Figure 1
		◦ Enrollement: the numbers of participants screended for eligibility, found to be eligible or not eligible, declined to be enrolled, and enrolled in the study	✓	Figure 1
		◦ Assigment: the numbers of participants assigned to a study condition	✓	Figure 1
		◦ Allocation and intevention exposure: the number of particpants assigned to each study condition and the number of participants who received each intervention	✓	Figure 1
		◦ Follow-up: the number of participants who completed the follow-up or did not complete the follow-up (i.e., lost to follow-up), by study condition	✓	Figure 1
		◦ Analysis: the number of participants included in or excluded from the main analysis, by study condiction	✓	Figure 1
		• Description of protocol deviations from study as planned, along with reasons	✓	Materials and Methods: patient selection
Recruitment	13	• Dates defining the periods of recruitment and follow-up	✓	Materials and Methods: patient selection
Baseline Data	14	• Baseline demographic and clinical characteristics of participants in each study condition	✓	Results: patients
		• Baseline characteristics for each study condition relevant to specific disease prevention research		not applicable
		• Baseline comparisions of those lost to follow-up and those retained, overall and by study condition		not done
		• Comparision between study population at baseline and target population of interest		not done
Baseline equivalence	15	• Data on study group equivalence at baseline and statistical methods used to control for bassline differences		not applicable

TREND Statement Checklist

Number analyzed	16	• Number of partipants (denominator) included in each analysis for each study condition, particulary when the denominators change for different outcomes; statement of the results in absolute numbers when feasible	✓	Results
		• Indication of whether the analysis strategy was "intention to treat" or, if not, description of how non-compliers were treated in the analysis	✓	Results
Outcomes and estimation	17	• For primary and secondary outcome, a summary of results for each estimation study condition, and the estimated effect size and a confidence inteval to indicate the precision	✓	Results
		• Inclusion of null and negative findings	✓	Results: response to gammaplex; pharmacokinetics analysis
		• Inclusion of results from testing pre-specified causal pathways through which the intervention was intended to operate, if any		not applicable
Ancillary analyses	18	• Summary of other analyses performed, including subgroup or restricted analyses, indicating which are pre-specified or exploratory	✓	Results: pharmacokinetics analysis
Adverse events	19	• Summary of all important adverse events or unintended effects in each study condition (including summary measures, effect size estimates, and confidence intervals)	✓	Results: safety and tolerability
**Discussions**				
Interpretation	20	• Interpretation of the results, taking into account study hypotheses, sources of potential bias, imprecision of measures, multiplicative analyses, and other limitations or weaknessess of the study	✓	Discusion
		• Discussion of results taking into account the mechanism by which the intervention was intended to work (causal pathways) or alternative mechanisms or explanations		Discusion
		• Discussion of success of an barriers to implemnting the intervention, fidelity of implementation	✓	Discusion
		• Discussion of research, programmatic, or policy implications	✓	Discusion
Generalizability	21	• Generalizability (external validity) of the trial findings, taking into account the study population, the characteristics of the intervention, length of follow-up, incentives, complance rates, specific sites/settings involved in the study, and other contextual issues	✓	Discusion
Overall Evidence	22	• General interpretation of the results in the context of current evidence and current theory	✓	Discusion

From: Des Jarlais, D.C., Lyles, Crepaz, N., & the Trend Group (2004). Improving the reporting quality of nonramdomized evaluations of behaviorial and public health interventions: the TREND statement.Americian Journal of Public Health, 94, 361-366. For more information, visit: http://www.cdc.gov/trendstatement/
