# Supplementary material for: Safety and Efficacy of Gammaplex® in Idiopathic Thrombocytopenic Purpura (ClinicalTrials.gov - NCT00504075)
Source: PLoS One. 2014 Jun 3;9(6):e96600. doi: 10.1371/journal.pone.0096600 (PMC4043496; doi:10.1371/journal.pone.0096600)
Supplement: Protocol S1 — Trial Protocol. (PDF) [file pone.0096600.s002.pdf]

CLINICAL STUDY PROTOCOL

A PHASE III, MULTICENTER, OPEN-LABEL STUDY TO EVALUATE THE EFFICACY AND SAFETY OF GAMMAPLEX® IN CHRONIC IDIOPATHIC THROMBOCYTOPENIC PURPURA

A Phase III Study

**Protocol No.**	**GMX02**
**Version**	**Version 7**
**Date**	**22 July 2009**
**Name of Investigational Product**	**GAMMAPLEX®**
**Name and Address of Sponsor**	**Bio Products Laboratory,<br>Dagger Lane, Elstree, Hertfordshire, U.K.**

NOTE: Information contained within this document is for use only by the intended recipient. It must be treated as confidential at all times. Do not disclose any information contained in this document to any person not involved in the study.

SIGNATURE PAGE

Sponsor's Medical Director

Dr. Clive Dash MB, ChB, FFPM
Bio Products Laboratory
Dagger Lane, Elstree
Hertfordshire, WD6 3BX, U.K.
Phone: +44 (0) 20 8258 2565
Fax: +44 (0) 20 8258 2611
Email: clive.dash@bpl.co.uk

C Dash 22 July 2009

Signature Date

TABLE OF CONTENTS

1. LIST OF ABBREVIATIONS AND DEFINITION OF TERMS 6

2. PROTOCOL SYNOPSIS 8

3. INTRODUCTION 12

3.1. BACKGROUND 12
3.2. BENEFIT/RISK STATEMENT 13

4. STUDY OBJECTIVES 13

4.1. PRIMARY OBJECTIVE 13
4.2. SECONDARY OBJECTIVE 13

5. STUDY DESIGN 14

5.1. STUDY ADMINISTRATION 14
5.1.1. Primary Investigator 14
5.1.2. Contract Research Organization 14
5.1.3. Central Laboratory 14
5.1.4. Distributor/Central Pharmacy 14
5.1.5. Sample Storage Facility 14
5.2. OVERALL STUDY DESIGN 14
5.3. STUDY VISITS 15
5.3.1. Description of Study Visits 16
5.3.1.1. Screening Visit 16
5.3.1.2. Infusion Visits for First Treatment Course (Days 1 and 2) 18
5.3.1.3. Follow-Up Visits for First Treatment Course (Days 3, 5, 9, 14, 21 and 32) 20
5.3.1.4. Infusion Visits for Additional Treatment Course(s) (Infusions 3–6) 21
5.3.1.5. Follow-Up Visits for Additional Treatment Course(s) (Infusions 3–6) 22
5.3.1.6. End of Study Visit 23

6. STUDY POPULATION 29

6.1. INCLUSION CRITERIA 29
6.2. EXCLUSION CRITERIA 29
6.3. SUBJECT WITHDRAWAL 31
6.4. STUDY TERMINATION 31

7. DESCRIPTION OF TREATMENT ADMINISTERED 31

7.1. INVESTIGATIONAL PRODUCT 31
7.1.1. Identification and Batch Numbers 31
7.1.2. Packaging and Labeling 32
7.1.3. Conditions for Storage and Use 32
7.2. INVESTIGATIONAL TREATMENT 32
7.2.1. Dose Schedule and Rationale 32

7.2.2. Product Preparation....32
7.2.3. Method of Administration....33
7.2.4. Product Accountability....34
7.3. TREATMENT COMPLIANCE....35

8. ASSESSMENT OF EFFICACY....35

8.1. PRIMARY EFFICACY VARIABLE....35
8.2. SECONDARY EFFICACY VARIABLES....35

9. ASSESSMENT OF SAFETY....35

9.1. SAFETY VARIABLES....35
9.1.1. Adverse Events....35
9.1.1.1. Definitions....36
9.1.1.2. Assessment of Severity....37
9.1.1.3. Assessment of Relationship....39
9.1.1.4. Follow-Up of Adverse Events....39
9.1.2. Submission of Serious Adverse Event Reports....39
9.1.3. Regulatory Reporting Requirements for Serious Adverse Events....40
9.1.3.1. Regulatory Reporting Requirements for SUSARS....41
9.2. VITAL SIGNS....41
9.3. CLINICAL LABORATORY TESTS AND DIRECT COOMBS' TEST....41
9.3.1. Clinical Laboratory Tests....41
9.3.1.1. Effects on Hepatic, Renal, and Hematologic Function....43
9.3.2. Direct Coombs' Test....43
9.4. VIRAL TRANSMISSION....43
9.5. PHYSICAL EXAMINATION....43

10. STATISTICAL METHODS....44

10.1. SAMPLE SIZE....44
10.2. ANALYSIS POPULATION....44
10.3. EFFICACY ANALYSES....44
10.3.1. Analysis of the Primary Efficacy Variable....44
10.3.2. Analysis of the Secondary Efficacy Variables....44
10.4. SAFETY ANALYSES....45
10.4.1. Adverse Events....45
10.4.2. Other Safety Assessments....45
10.5. INTERIM/PARTIAL ANALYSES....45
10.5.1. Interim Analysis....45
10.5.2. Partial Analysis....46

11. ETHICAL AND REGULATORY COMPLIANCE....46

11.1. ETHICAL / REGULATORY FRAMEWORK....46

11.2. INSTITUTIONAL REVIEW BOARD 46
11.3. SUBJECT INFORMATION AND INFORMED CONSENT 46
12. QUALITY CONTROL AND QUALITY ASSURANCE 47
12.1. SOURCE DATA AND RECORDS 47
12.2. PERIODIC MONITORING 47
12.3. AUDIT AND INSPECTION 47
12.4. CONFIDENTIALITY OF SUBJECTS' DATA 47
13. DATA HANDLING AND RECORD KEEPING 48
13.1. CASE REPORT FORMS 48
13.1.1. Changes to Case Report Form Data 48
13.2. INVESTIGATOR FILE 48
13.3. PROVISION OF ADDITIONAL INFORMATION 48
14. CHANGES IN THE CONDUCT OF THE STUDY 49
14.1. PROTOCOL AMENDMENTS 49
14.2. PREMATURE STUDY TERMINATION 49
15. REPORTING AND PUBLICATION 49
15.1. STUDY REPORTS 49
15.1.1. Interim Clinical Study Report 49
15.1.2. Final Clinical Study Report 49
15.2. PUBLICATION POLICY 49
16. REFERENCES 50

List of In-Text Figures and Tables

Figure 1 Study Flow Chart 25
Table 1 Schedule of Assessments 26
Table 2 Additional Assessments for Subjects who Require 2 or 3 Courses of GAMMAPLEX (Infusions 3 - 6) 28
Table 3 Starting, Incremental, and Maximum Infusion Rates 34
Table 4 Criteria for Grading Severity of Infusion-Related Adverse Events[a] that Occur During Infusion or up to 60 minutes from the End of Infusion 38

List of Appendixes

Appendix 1: Assessment of Bleeding/Hemorrhage 52
Appendix 2: Investigator Agreement 55

1. LIST OF ABBREVIATIONS AND DEFINITION OF TERMS

AEs	Adverse events
ALT	Alanine transaminase
ANC	Absolute neutrophil count
AST	Aspartate transaminase
BPL	Bio Products Laboratory (Sponsor/Manufacturer)
BUN	Blood urea nitrogen
CBC	Complete blood count
CNS	Central nervous system
CPMP	Committee for Proprietary Medicinal Products
CRA	Clinical Research Associate
CRF	Case Report Form
CRO	Contract Research Organization
CVA	Cardiovascular accident
DBP	Diastolic blood pressure
DCT	Direct Coombs' Test
DVT	Deep vein thrombosis
EMEA	European Medicines Agency
EU	European Union
FDA	U.S. Food & Drug Administration
GI	Gastrointestinal
HAV	Hepatitis A virus
HBsAg	Hepatitis B surface antigen
HBV	Hepatitis B virus
HCG	Human chorionic gonadotrophin
HCV	Hepatitis C virus
HIV	Human Immunodeficiency Virus
ICH GCP	International Conference on Harmonization Good Clinical Practice
IgA	Immunoglobulin A
IgG	Immunoglobulin G
IGIV	Immunoglobulin Intravenous
IgM	Immunoglobulin M
INC	INC Research (CRO)
IRB/IEC	Institutional Review Board (U.S.)/Independent Ethics Committee (India) (also refers to Comité Institucional de Etica [Argentina])
ITP	Idiopathic Thrombocytopenic Purpura
ITT	Intent-to-treat (population)
IV	Intravenous
LDH	Lactic dehydrogenase
min	Minute
mmHg	Millimeters of mercury
mOsmol	MilliOsmolal
NAT	Nucleic acid amplification test (PCR)
NCR	Non-carbon reproduced
P_0	The historical response rate (lower bound of 95% confidence interval)

P_1	Response rate at time of the partial analysis
PCR	Polymerase chain reaction
PK	Pharmacokinetics
PVC	Polyvinyl chloride
RBC	Red blood cell count
Rh(D)	Rhesus D
RNA	Ribonucleic acid
SAE	Serious adverse event
SOP	Standard operating procedure
SUSAR	Suspected unexpected SAE
TEAV	Treatment-emergent abnormal value
TIA	Transient ischemic attack
TKO	To keep open
U.K.	United Kingdom
U.S.	United States

2. PROTOCOL SYNOPSIS

**Name of Sponsor/Company:**	**Name of Manufacturer:**
Bio Products Laboratory (BPL)<br>Dagger Lane, Elstree<br>Hertfordshire, WD6 3BX. U.K.	Bio Products Laboratory (BPL)<br>Dagger Lane, Elstree<br>Hertfordshire, WD6 3BX. U.K.
**Name of Finished Product:**<br>GAMMAPLEX®	**Protocol identification code:**<br>GMX02
**Name of Active Ingredient:**<br>Immune Globulin (Human)	**Protocol Version and Date:**<br>Version 7, 22 July 2009

Title of study: A Phase III, Multicenter, Open-Label Study To Evaluate the Efficacy and Safety of GAMMAPLEX® in Chronic Idiopathic Thrombocytopenic Purpura

Indication: Chronic idiopathic (immune) thrombocytopenic purpura (platelet count $\leq 20 \times 10^9/L$ at enrollment)

Study centers: Approximately 25 sites in the U.S., India, and Argentina

Development phase: III

Objectives: The primary objective is to determine if BPL's GAMMAPLEX raises the platelet count of subjects with chronic ITP to a threshold of $50 \times 10^9/L$, similar to that of a historical control. The secondary objectives are: **1)** to determine the safety of GAMMAPLEX at the dosage used in this study. **2)** to determine if GAMMAPLEX maintains platelet counts of $\geq 50 \times 10^9/L$ in subjects with chronic ITP for a period of time similar to that of a historical control.

Study design: This is a multicenter open label study of the efficacy and safety of GAMMAPLEX. After screening and enrollment, subjects will receive infusions of GAMMAPLEX on Days 1 and 2 (first treatment course). Subjects will be assessed for safety and efficacy on Days 3, 5, 9, 14, 21, and 32; they will then return for a final follow-up visit (End of Study visit), to include viral safety tests on Day 90.

If the platelet count is not maintained for the desired length of time after the first course of GAMMAPLEX, and at the discretion of the study investigator and subject/guardian, subjects may receive up to a further 2 courses of GAMMAPLEX within the period Day 32 to Day 90 of the first treatment course. Subjects will have a follow-up visit 3 days after the second infusion in each treatment course. The End of Study visit for subjects receiving an additional 1 or 2 courses of GAMMAPLEX will take place 90 days, approximately 3 months, after the first infusion of the last course of GAMMAPLEX.

For all subjects the End of Study visit will be approximately 3 months after the last dose of GAMMAPLEX and will be the End of Study visit for all subjects irrespective of whether they received 1, 2, or 3 courses. For subjects receiving 1 treatment course of GAMMAPLEX only, this visit will take place on Day 90.

There may be an interim analysis performed during the study. This may be performed when at least 30 evaluable adult subjects have completed Day 32. All other subjects recruited up to this time point, e.g. non-evaluable subjects or children (applicable to subjects enrolled before implementation of Protocol Version 7) will be analyzed for safety only. The purpose of this analysis is to facilitate submission of an application to the U.K. and EU regulatory authorities.

A further partial analysis may be performed when at least 27 evaluable subjects have completed Day 9. The

**Name of Sponsor/Company:**<br>Bio Products Laboratory (BPL)<br>Dagger Lane, Elstree<br>Hertfordshire, WD6 3BX. U.K.	**Name of Manufacturer:**<br>Bio Products Laboratory (BPL)<br>Dagger Lane, Elstree<br>Hertfordshire, WD6 3BX. U.K.
**Name of Finished Product:**<br>GAMMAPLEX®	**Protocol identification code:**<br>GMX02
**Name of Active Ingredient:**<br>Immune Globulin (Human)	**Protocol Version and Date:**<br>Version 7, 22 July 2009

observed response rate will be estimated and compared with the historical control of >0.60 to re-estimate the sample size.

Number of subjects: A minimum of 31 evaluable subjects will be enrolled in the study. The final number will depend on the number estimated by the power calculation in the partial analysis to be performed when at least 27 evaluable subjects have completed Day 9.

Study variables: Efficacy:

Efficacy will be assessed for the first course of GAMMAPLEX only.

The primary efficacy variable will be the percent of subjects attaining a platelet count of ≥50 x 10^9/L by Day 9 (the 7th day after completing the second infusion). There will be 2 secondary efficacy variables: Duration of time for which the platelet counts remain ≥50 x 10^9/L after the first course of GAMMAPLEX and changes in the signs of any bleeding/hemorrhage up to Day 32.

Safety: The variables used to assess safety will be the following: adverse events; vital signs; clinical laboratory tests and Direct Coombs' Test; any transmission of viruses; physical examination.

Methodology: Efficacy: The primary efficacy variable will be analyzed using the intent–to-treat population (ITT). The percent of subjects attaining a platelet count of ≥50 x 10^9/L by Day 9 will be compared with the historical response rate of >60%. A 1-sided 95% confidence interval will be constructed for the percent of subjects attaining a platelet count of ≥50 x 10^9/L by Day 9 for GAMMAPLEX. If the lower bound of this confidence interval is >0.60, GAMMAPLEX will be considered effective for the treatment of chronic ITP.

The secondary efficacy variables will be analyzed by: 1) using the method of Kaplan and Meier to produce plots of the distribution of the time for which the platelet counts remain ≥50 x 10^9/L; 2) a 95% confidence interval for the median time will be constructed.

Bleeding/hemorrhage events will also be summarized by number and percent (Appendix 1).

Safety: The proportion of infusions where an AE occurs during an infusion or within 72 hours after the end of the last infusion (Day 2) will be tabulated. A summary table, including number of subjects with AEs, SAEs, deaths, withdrawn due to AEs, and product-related AEs will be produced. The nature, severity and frequency of all AEs will be listed.

Summary tables for laboratory measures of hepatic, renal, and hematologic function will include the following: descriptive statistics (mean, standard deviation, median, maximum and minimum) for original values and change from baseline for all continuous variables; incidence (number and percent) of clinically significant, treatment-emergent abnormal values (TEAVs); shift tables will be used to show the incidence of new or worsening clinically

**Name of Sponsor/Company:**<br>Bio Products Laboratory (BPL)<br>Dagger Lane, Elstree<br>Hertfordshire, WD6 3BX. U.K.	**Name of Manufacturer:**<br>Bio Products Laboratory (BPL)<br>Dagger Lane, Elstree<br>Hertfordshire, WD6 3BX. U.K.
**Name of Finished Product:**<br>GAMMAPLEX®	**Protocol identification code:**<br>GMX02
**Name of Active Ingredient:**<br>Immune Globulin (Human)	**Protocol Version and Date:**<br>Version 7, 22 July 2009

significant findings from baseline.

The proportion of subjects in whom there is a transmission of Hepatitis B, Hepatitis C, HIV, or Parvovirus B19 will be tabulated. The results from the Direct Coombs' Test will also be tabulated.

All abnormalities of physical assessment after baseline will be summarized by using number and percent. Shift tables from normal to abnormal and abnormal to normal will also be produced.

Summary statistics mean, median, standard deviation, maximum and minimum of original values and change from baseline will be provided for all vital signs measured – diastolic and systolic blood pressure (mmHg), pulse rate (beats/min), respiration rate (breaths/min) and oral temperature ($^{\circ}$C).

Subject selection criteria: **Inclusion Criteria:**

1) Males and females aged between 18 and 70 years. **2)** Confirmed diagnosis of chronic ITP of at least 6 months duration. **3)** Platelet count $\leq 20 \times 10^9$/L at enrollment. **4)** Absence of other conditions that, in the opinion of the investigator, could cause thrombocytopenia. **5)** If subjects are currently being treated with corticosteroids the treatment regimen/dose must have been stable (for a minimum of 2 weeks before Day 1 infusion). However, subjects must remain on a stable treatment regimen. If there is any intent to alter the corticosteroid treatment regimen (e.g., tapering of corticosteroids) before Day 10, subjects may not be included in the study. **6)** If subjects are currently being treated with cyclophosphamide, azathioprine or attenuated androgens, the treatment regimen and dose must have been stable for a minimum of 2 months before infusion on Day 1. However, if there is any intent to alter the treatment regimen before Day 10, subjects may not be included in the study. **7)** Splenectomized subjects and both Rh(D)+ and Rh(D)- subjects may be included. **8)** The subject has signed an informed consent form (subjects must be at least 18 years old), and/or the subject's legal guardian has signed the informed consent form if indicated. **9)** If a subject is a female of child-bearing potential, she must have a negative result on a urine-based HCG pregnancy test. **10)** If a subject is a female who is or becomes sexually active, she must practice contraception by using a method of proven reliability for the duration of the study.

Exclusion Criteria: 1) The subject has a history of any severe or anaphylactic reaction to blood or any blood-derived product, or any severe reaction to IGIV or any other IgG preparation. **2)** The subject is known to be intolerant to any component of the investigational product. **3)** The subject has received any live virus vaccine within the last 3 months prior to Day 1. **4)** The subject has received an IGIV preparation within 1 month prior to Day 1. **5)** The subject is currently receiving, or has received, any investigational agent within the 1 month prior to Day 1. **6)** The subject has received any blood, blood product, or blood derivative within the 1 month prior to Day 1. **7)** The subject has received Rituximab within the 3 months before Day 1. **8)** The subject is pregnant or is nursing. **9)** The subject is positive for any of the following at screening: HBsAg, NAT for HCV, NAT for HIV,

**Name of Sponsor/Company:** Bio Products Laboratory (BPL) Dagger Lane, Elstree Hertfordshire, WD6 3BX. U.K.	**Name of Manufacturer:** Bio Products Laboratory (BPL) Dagger Lane, Elstree Hertfordshire, WD6 3BX. U.K.
**Name of Finished Product:** GAMMAPLEX®	**Protocol identification code:** GMX02
**Name of Active Ingredient:** Immune Globulin (Human)	**Protocol Version and Date:** Version 7, 22 July 2009

Antibodies to HCV or HIV 1 or 2. **10)** The subject, at screening, has levels greater than 2.5 times the upper limit of normal, as defined by the central laboratory, of alanine aminotransferase or aspartate aminotransferase. **11)** The subject has a severe renal impairment (defined as serum creatinine greater than 2 times the upper limit of normal or BUN greater than 2.5 times the upper limit of normal for the range of the laboratory doing the analysis); the subject is on dialysis; the subject has a history of acute renal failure. **12)** The subject is known to have abused alcohol, opiates, psychotropic agents, or other chemicals or drugs within the past 12 months. **13)** The subject has a history of deep vein thrombosis (DVT) or thrombotic complications of IGIV therapy. **14** The subject has any history or sign of hyperviscosity, transient ischemic attack (TIA), stroke, other thromboembolic event, or unstable angina. **15)** The subject suffers from any acute or chronic medical conditions (e.g., renal disease or predisposing conditions for renal disease, coronary artery disease, or protein losing enteropathy) that, in the opinion of the investigator, may interfere with the conduct of the study. **16)** The subject has an acquired medical condition, such as chronic lymphocytic leukemia, lymphoma, multiple myeloma, chronic or recurrent neutropenia (defined as an absolute neutrophil count (ANC) < 1 x 10^9/L). **17)** The subject has non-controlled arterial hypertension (systolic blood pressure >160 mmHg and/or diastolic blood pressure >90 mmHg). **18)** The subject is anemic (hemoglobin <10 g/dL) at screening. **19)** The subject is unlikely to adhere to the protocol requirements of the study or is likely to be uncooperative.

Test product, dose/mode of administration, batch number(s): The first course of GAMMAPLEX will be administered as an intravenous infusion of 1 g/kg on each of 2 consecutive days. If required, a further 1 or 2 courses on the same dosage regimen may be administered in the period Day 32 to Day 90 following the first course of GAMMAPLEX.

At least 2 batches of GAMMAPLEX will be used in this study.

Duration of treatment: Each subject can receive up to 3, 2-day courses of GAMMAPLEX over a period of up to 90 days. The total duration of the study for each subject will be 90 days after the first infusion of the last course of GAMMAPLEX which could range from 90–180 days (approximately 3 to 6 months) depending on the number and timing of treatment courses received.

3. INTRODUCTION

3.1. Background

Idiopathic (immune) thrombocytopenic purpura (ITP) is an autoimmune disorder affecting both children and adults; it is characterized by a low platelet count, normal results on a bone marrow examination (except possibly for increased megakaryocytes), and the absence of specific causes of thrombocytopenia, such as leukemia, aplastic anemia or disseminated intravascular coagulation [1-3]. Childhood ITP is typically of acute onset. In more than 70% of children, spontaneous and permanent remission occurs within 1 year of onset [3,4]. In contrast, the majority of adults have persistent ITP, although the natural history is less defined than that for ITP in children, and some patients do improve with time [5,6]. Rarely, life-threatening bleeding occurs, but when it does, intracranial hemorrhage is the principal cause of death [7,8]. Serious hemorrhage is most likely to occur when the platelet count falls below 20 x 10^9/L [2,3,5]. Therefore, treatment options for ITP are intended rapidly to increase the platelet count and include glucocorticoids, immune globulin intravenous (IGIV) and, in $Rh(D)^+$ patients, intravenous anti-D (Rho) globulin [2].

The first report of the efficacy of IGIV in the treatment of ITP appeared in 1981 [9]. More than 100 studies have subsequently confirmed the safety and efficacy of IGIV as treatment of ITP in children and adults [10-15]. A substantial and rapid platelet increase can be achieved with an IGIV dosage of 1 g/kg/day repeated for 2 consecutive days and this is now preferred to the original regimen of 400 mg/kg/day repeated daily for 5 days [15-17].

GAMMAPLEX® 5% is a newly developed, highly purified, unmodified IgG product intended for intravenous (IV) administration; it is manufactured by Bio Products Laboratory (BPL), United Kingdom (U.K.) from plasma from healthy U.S. donors who are subjected to medical examinations, laboratory tests, and a review of their medical history. Each donation must be non-reactive for Hepatitis B surface antigen (HBsAg), anti-HIV-1 and HIV-2 antibodies, and anti-HCV antibodies. Furthermore, plasma minipools (512 donations per pool) undergo nucleic acid amplification/polymerase chain reaction (NAT/PCR) testing for HIV, HBV, HCV, Hepatitis A (HAV), and Parvovirus B19. Manufacturing pools are tested for HBsAg, HIV-1/2 antibodies; HCV and Parvovirus B19 are also tested using NAT/PCR.

GAMMAPLEX is a ready-prepared solution for IV administration that contains 5 g human normal immunoglobulin and 5 g D-sorbitol (as stabilizer) in 100 mL of buffer solution containing: 0.6 g glycine, 0.2 g sodium acetate, 0.3 g sodium chloride, and ~5 mg Polysorbate 80. Immunoglobulin G purity reaches 100%, the pH is in the range of 4.8 to 5.0, and osmolality is not less than 240 mOsmol/kg (typically 420 to 500 mOsmol/kg). The immunoglobulins present are virtually 100% IgG, and the distribution of the four IgG subclasses is approximately 62% IgG_1, 31% IgG_2, 6% IgG_3, and 1% IgG_4. The content of IgA is lower than 10 µg/mL. The Anti-D content of the final product is monitored and controlled to specification [18].

In a preclinical study in male Wistar rats, some small, but statistically significant, hypertensive differences were seen in the GAMMAPLEX group when compared with the vehicle control group after 140 minutes. These differences were not seen in another study when the product was infused at a lower rate; it was concluded that the rise in blood pressure with GAMMAPLEX in the first study was due to infusion rate, the total dose of IgG infused, or both. Furthermore, the

infusion rates used in these nonclinical studies convert to a clinical rate of 0.07-0.1 mL/kg/min. The recommended starting rate for the infusions in subjects receiving GAMMAPLEX is 0.01-0.02 mL/kg/min, so hypertension should not be an issue in clinical studies [19].

GAMMAPLEX 400 mg/kg IGIV given at up to 6 mL/min was well tolerated in a single-dose pharmacokinetics (PK) study in 36 healthy volunteers. No SAEs were reported [20].

3.2. Benefit/Risk Statement

ITP is potentially fatal mainly because of the risk of intracranial hemorrhage. Other bleeding may occur and may result in acute or chronic anemia. The use of IGIV to rapidly increase platelet count has been shown in other studies to decrease signs and symptoms of bleeding/hemorrhage and may obviate or delay the need for splenectomy. Splenectomy carries an intraoperative risk of hemorrhage and the long term risk to the patient of infections (especially pneumococcal) as well as failure to permanently increase platelet counts.

Most of the IGIV used in the U.S. is for off-label indications, such as peripheral neuropathy and other autoimmune diseases. In addition, there have been several mergers of manufacturers of IGIV, resulting in fewer products on the market. Although the manufacturers have generally kept up with the demand for IGIV, these changes in the marketplace have resulted in disruption in the supply of different products at different times. Changes in products may result in an increased frequency of adverse events (AEs) in patients who previously tolerated a given preparation [19]. For this reason, entry of additional manufacturers into the U.S. market (and potentially other markets) may help to provide a steady stream of consistent products for ITP patients.

The European Medicines Agency (EMEA) regards ITP as a good model for other conditions with a known or presumed immunological pathogenesis, because this condition tests out the integrity/activity of the Fc structure of the IgG molecule.

As with any blood product, viral safety is a concern. GAMMAPLEX undergoes a stringent multi-step process, including solvent/detergent treatment, nanofiltration and a terminal low pH incubation of the finished product, to reduce the risk of potential viral contaminants and enhance the safety of the product [19].

IGIV products have been associated with renal AEs, particularly in those products containing sucrose as a stabilizer. GAMMAPLEX contains sorbitol as a stabilizer, thereby reducing the risk of such reactions.

4. STUDY OBJECTIVES

4.1. Primary Objective

The primary objective is to determine if BPL's GAMMAPLEX raises the platelet count of subjects with chronic ITP to a threshold of 50 x 10^9/L, similar to that of a historical control [9-17,21].

4.2. Secondary Objective

The secondary objectives are:

- To determine the safety of GAMMAPLEX at the dose used in this study.

- To determine if GAMMAPLEX maintains platelet counts of ≥ 50 x 10^9/L in subjects with chronic ITP for a period of time similar to that of a historical control [9-17,21].

5. STUDY DESIGN

5.1. Study Administration

5.1.1. Primary Investigator

The primary investigator at each site is responsible for all functions performed at their site as a part of this study. Qualified personnel who also perform protocol-defined functions will be identified in a Study Personnel Signature Log that will be maintained at each site.

5.1.2. Contract Research Organization

The contract research organizations (CROs) in this study are:

U.S.

INC Research, Inc. 4700 Falls of Neuse Road, Suite 400, Raleigh, NC 27609, U.S.A.

Argentina

Activa-Cro, Santa Fe 1592 - Piso 4°, C1060ABO, Buenos Aires, Argentina.

India

INC GVKBIO Pvt Ltd, 510-511 JMD Pacific Square, 32nd Milestone, Sector 15, Part II

Gurgaon – 122 001, Haryana, India

INC GVKBIO will be operating with INC Research, Inc.

5.1.3. Central Laboratory

All samples for laboratory tests should be shipped to the selected central laboratory in each country for the study, as described in the Laboratory Manual.

5.1.4. Distributor/Central Pharmacy

Study product will be exported to and will be stored by the selected central distributor/pharmacy in each country and will be distributed to the investigator site as described in the Pharmacy Manual.

5.1.5. Sample Storage Facility

Retention samples (see Section 9.3.1) will initially be shipped from the investigator site to the central laboratory, as detailed in the laboratory manual. Those samples will later be shipped by the central laboratory to Cryo-store, Greenwich Centre Business Park, London, U.K, for long-term storage.

5.2. Overall Study Design

This will be a multicenter, open-label study conducted at approximately 25 clinical sites in the U.S., India, and Argentina. A minimum of 31 evaluable subjects will be enrolled in the study.

The final number will depend on the number estimated by the power calculation in the partial analysis.

After screening and enrollment, subjects will initially receive infusions of GAMMAPLEX on Days 1 and 2 (first treatment course). Subjects will be assessed for safety and efficacy on Days 3, 5, 9, 14, 21, and 32; then they will then return for a final follow–up visit (End of Study), to include viral safety tests on Day 90.

If the platelet count is not maintained for the desired length of time after the first course of GAMMAPLEX, and at the discretion of the study investigator and subject/guardian, subjects may receive up to 2 additional courses of GAMMAPLEX within the 32 to 90 days after the first dose. Subjects who receive additional treatments will attend all safety and efficacy follow-up visits as scheduled for the first treatment course. In addition, they will be assessed for safety prior to each additional dose of GAMMAPLEX and 3 days after the end of each treatment course. All subjects will attend one End of Study visit, which will be 90 days, approximately 3 months, after the first infusion of the last course of GAMMAPLEX (see Section 5.3.1.6).

Efficacy data will be collected and analyzed for the first course of GAMMAPLEX only.

5.3. Study Visits

The study visits are summarized in the study flow chart (Figure 1). The schedule of assessments is in Table 1, and in Table 2 for subjects who receive additional treatment courses. The study visits are identified as follows:

- Screening visit (can occur up to 6 months prior to Day 1)
- Visit(s) to check platelet count, (if applicable) – for those subjects whose platelet count is above 20 x 10^9/L at the screening visit, samples can be sent to the local laboratory to check the platelet count. For subjects screened more than 14 days prior to Day 1, additional safety laboratory samples will be analyzed by the local laboratory e.g. CBC, LFTs, serum Creatinine/BUN.
- First course of GAMMAPLEX – there will be 2 infusion visits on consecutive days (Days 1 and 2)
- Follow-up – 6 follow-up visits from Day 3 to Day 32
- Second and third courses of GAMMAPLEX – at the discretion of the investigator, subjects may receive 1 or 2 more courses of GAMMAPLEX within the period 32 to 90 days following the first course of GAMMAPLEX. Each course will consist of 2 infusions on consecutive days and will be scheduled at an appropriate time following the first treatment course, depending upon the subject's response to the previous treatment course of GAMMAPLEX
- End of Study visit – For all subjects this visit will be approximately 3 months after the first infusion of the last course of GAMMAPLEX and will be the End of Study visit for all subjects irrespective of whether they received 1, 2, or 3 courses. For subjects receiving 1 treatment course of GAMMAPLEX only, this visit will take place on Day 90.

5.3.1. Description of Study Visits

5.3.1.1. Screening Visit

Subject Eligibility

The subject's eligibility to participate in the study will be assessed at this visit according to the inclusion and exclusion criteria. This visit can occur up to 6 months prior to Day 1.

Informed Consent

Written informed consent must be obtained from the subject by qualified personnel and signed by the investigator before any study procedures, including screening laboratory tests, are carried out (see Section 11.3). Informed consent can be taken up to 6 months prior to Day 1.

Pregnancy Test

All female subjects of childbearing potential will receive a urine-based, human chorionic gonadotrophin (HCG) pregnancy test irrespective of claimed inability to conceive.

Complete Medical History

A complete medical history will be obtained from each subject at this visit. The history will specifically include documentation of the diagnosis of chronic ITP, the reason why other causes of thrombocytopenia were excluded, general medical history, history and outcome of previous use of and/or reactions to any IGIV or other blood product, history and treatment of ITP, and history of bleeding/hemorrhage. Bone marrow examination is not required, but if it has been done, it should be consistent with ITP with, for example, normal or increased megakaryocytes. For subjects who have had bone marrow examinations, the date and findings should be recorded on the Case Report Form (CRF) at the time of screening.

The medical history must include the use of previous IGIV treatments received; the product name, the unit dose, and how they were tolerated must be recorded. The previous use of an IGIV product must be documented in the subject's CRF. In addition the treatment regimen and dose of corticosteroids, azathioprine, cyclophosphamide, and attenuated androgens must be documented in the subject's CRF.

Physical Examination

Physical examination will be performed by appropriately qualified personnel. The physical examination is also a safety variable (see Section 9.5.).

Vital Signs

Vital signs assessed by qualified personnel will be entered on the CRF. The vital signs to be observed will be diastolic and systolic blood pressure (mmHg), pulse rate (beats/min), respiration rate (breaths/min), and oral temperature (°C). The subject must be in the same position each time vital signs are obtained. The vital signs are also a safety variable (see Section 9.2.).

Blood Samples for Laboratory Tests

Blood samples will be obtained for Laboratory A and Laboratory B (Clinical Laboratory tests are a safety variable, see Section 9.3). If a subject is otherwise eligible, but the platelet count is greater than 20 x 10^9/L, the subject will not be enrolled. If at a subsequent visit (within 6 months of the screening visit) the subject's clinical condition suggests to the investigator that their platelet count may have dropped, the subject may have their platelet count re-measured by the local laboratory. If the platelet count is $\leq$ 20 x 10^9/L, the general health of the subject has not significantly changed, and no changes in concomitant medications or treatment have occurred which would violate the inclusion criteria, then safety blood samples should be measured by the local laboratory e.g. CBC, LFTs, serum Creatinine/BUN. If these all meet the entry criteria then the subject can be considered as eligible, and can be immediately enrolled in the study.

Other Blood Samples

The following additional samples will be collected:

- Serum for IgG, IgA, and IgM levels
- A reserve sample (see Section 9.3.1)
- A sample for a Direct Coombs' test (see Section 9.3.2).
- NAT for HCV, HIV and Parvovirus B19
- Serology for HIV_1 and HIV_2, HCV, HBsAg
- Blood typing (A, B, O, $Rh(D)^{+/-}$)

Urinalysis

A urinalysis with microscopic examination will be done as a part of this visit.

Assessment for Bleeding/Hemorrhage

The subject will be assessed for bleeding and hemorrhage; this will be graded as detailed in Appendix 1 and recorded on the subject's CRF.

Concomitant medications

If the subject is enrolled within 14 days of screening, all medications the subject is taking at the time of screening may be continued through the completion of the second infusion, including corticosteroids; however, the dose must remain unaltered through Day 9 unless some condition of the subject requires a change. If possible, doses of corticosteroids or other immunosuppressant medications should remain constant until Day 32 (see Section 6.1). All concomitant medications will be recorded on the CRFs.

If the subject is not enrolled within 14 days of screening, it should be noted that doses/regimen of corticosteroid must have been stable for 2 weeks prior to Day 1; and doses/regimen of azathioprine, cyclophosphamide, and attenuated androgens must have been stable for 2 months prior to Day 1. Changes to the dosage of these medications should be avoided as mentioned above. In addition subjects are not to receive an IGIV preparation or an investigational agent or

any blood, blood product or blood derivative within 1 month prior to Day 1, and receipt of any live virus vaccine and Rituximab is not allowed within 3 months prior to Day 1.

Administration of corticosteroids to decrease adverse reactions to the infusion may not be given before the IGIV infusions. However, pre-medication with analgesic or antihistamine drugs is permissible if required.

No blood products, including IGIV (other than GAMMAPLEX in accordance with the protocol), may be given during the study until the End of Study visit (viral safety visit approximately 3 months after the last dose of GAMMAPLEX).

5.3.1.2. Infusion Visits for First Treatment Course (Days 1 and 2)

At these visits, the following will be done:

Eligibility and Enrollment

For subjects screened more than 14 days prior to Day 1, in addition to the sample taken to check the platelet count, blood samples for safety assessment by the local laboratory should be taken to ensure there have been no further changes in a subject's safety profile since screening, e.g. CBC, LFTs, serum Creatinine/BUN.

On Day 1 only, a sample for platelet count will be analyzed locally before the first infusion to ensure that the subject's platelet count is $\leq 20 \times 10^9$/L and that they are still eligible to enter the study.

The subject's eligibility to participate in the study will be confirmed only on Day 1. If eligible, the subject will be assigned a subject number. Subject numbers will be assigned as indicated in the Data Entry Instructions.

Samples for Laboratory A and B will be taken for testing by the central laboratory prior to infusion with GAMMAPLEX on Day 1 only. Eligibility to be included in the efficacy analysis will only be confirmed once the safety laboratory results are returned from the central laboratory.

Pregnancy Test

A urine-based HCG test will be done before the infusion with GAMMAPLEX on Day 1.

Interval Medical History

An interval medical history will be performed before each infusion. Any changes from screening will be recorded on the CRF.

Physical Examination

A physical examination will be performed before each infusion and the results recorded on the CRF.

Vital Signs

At each infusion visit, vital signs will be assessed by qualified personnel and entered in the CRF.

Vital signs will be recorded at the following times:

- 10 minutes before, and at the start of each infusion (to have 2 baselines)
- 10 minutes after initiation of the infusion, and 10 minutes after each rate increase
- 10 minutes and 30 minutes after the maximum rate is achieved, and every 60 minutes thereafter until the infusion is stopped
- the time at which each infusion is stopped
- 15 and 30 minutes after stopping each infusion

Before listing changes in vital signs as AEs, each change should be assessed for clinical relevance (see Sections 9.1.1.2 and 9.2). If AEs occur, vital signs will be monitored more frequently, as appropriate.

Blood Samples for Laboratory Tests

Samples for Laboratory A and B will be analyzed at the central laboratory.

Blood samples for Laboratory A will be drawn on Day 1 and Day 2 prior to infusion with study medication.

The following samples will also be drawn on Days 1 and 2 prior to infusion with study medication:

- A reserve sample (see Section 9.3.1)
- Serum for IgG levels

The following will be drawn on Day 1 only prior to infusion with study medication:

- Laboratory B
- A retention sample (see Section 9.3.1)
- A sample for a Direct Coombs' Test on Day 1 only (see Section 9.3.2)
- NAT for HCV, HIV and Parvovirus B19 on Day 1 only
- Serology for HIV_1 and HIV_2, HCV, and HBsAg on Day 1 only

Urinalysis

A urine sample for urinalysis on Day 1 only

Adverse Events

All AEs reported by the subject, recorded on the diary card or observed by the investigator will be recorded (Section 9.1.1).

Assessment for Bleeding/Hemorrhage

At each visit, the subject will be assessed for bleeding and hemorrhage; this will be graded as detailed in Appendix 1 and recorded in the subject's CRF (along with any bleeding episodes recorded in the subject's diary card).

Concomitant medications

All concomitant medications, including any entries on the diary cards, must be recorded in the subject's CRF including corticosteroids and immunosuppressants, if applicable (see also Section 5.3.1.1).

Diary Card

Each subject will be provided with a diary card at the first infusion visit. Subjects will be instructed to complete a diary entry every day from Day 1 until Day 32 describing any AEs and concomitant medications. Subjects should also document in their diaries any bleeding episodes, or the absence of such episodes, every day.

At each visit from Day 1 to Day 32, the study nurse will review the diary cards, and details of AEs, bleeding episodes, and concomitant medications will be entered in the subject's CRF. The study nurse will collect diaries at the Day 32 follow-up visit.

GAMMAPLEX Infusion

Each subject will be treated with GAMMAPLEX at a total dose of 2 g/kg body weight divided over 2 consecutive days (i.e., 1 g/kg on Day 1 and 1 g/kg on Day 2). IGIV will be infused by means of an infusion pump for precise infusion rates. (See Section 7.2 for details on treatment, dosing, and administration).

At the discretion of the investigator, if the subject is capable, he/she will be allowed to leave the study center after the completion of the assessment 60 minutes after the infusion.

5.3.1.3. Follow-Up Visits for First Treatment Course (Days 3, 5, 9, 14, 21 and 32)

At each visit the following will be done:

Interval Medical History

Any changes from the previous medical history on Day 1 should be recorded in the CRF.

Physical Examination

A physical examination will be performed at every visit and the results recorded in the CRF.

Vital Signs

Vital signs assessed by qualified personnel will be entered in the CRF at the Visits on Days 3, 5, and 9 only.

Blood Samples for Laboratory Tests

- Blood samples for Laboratory A will be drawn at each of the follow-up visits (Days 3, 5, 9, 14, 21, and 32).
- Samples for Laboratory B will be drawn on Days 3 and 9 and Day 32.
- Serum for IgG levels will be drawn at each visit
- Samples for Direct Coombs' Test will be taken on Day 5 and Day 14. (see Section 9.3.2). Samples for LDH, haptoglobin, and urine hemosiderin will also be taken on Day 5 and Day 14.

- NAT for Parvovirus B19 on Day 9 and Day 32.
- NAT for HCV and HIV on Day 32.
- Serology for HIV_1 and HIV_2, HCV, HBsAg on Day 32. Any seroconversion should be followed up.
- A reserve sample will be taken at each visit

Urinalysis

A urine sample for urinalysis will be taken on Days 3, 9, and 32.

Adverse Events

All AEs reported by the subject, recorded on the diary cards, or observed by the investigator will be recorded at these visits (see Section 9.1.1). On Day 5, particular attention should be paid to accurately collecting and recording all AEs that occur in the first 72 h after completion of the treatment course (see Section 10.4.1).

Assessment for Bleeding/hemorrhage

See Section 5.3.1.2.

Concomitant medications

Concomitant medications will be recorded at these visits, including entries on the diary cards. See Sections 5.3.1.1 and 5.3.1.2.

Diary Card:

At each visit, diary cards will be reviewed, and details of AEs, bleeding episodes, and concomitant medications will be entered on the CRF. See above and Section 5.3.1.2.

5.3.1.4. Infusion Visits for Additional Treatment Course(s) (Infusions 3–6)

Additional treatment courses are not mandatory, but they can be considered if the platelet count drops below 25 x 10^9/L. If the study physician/investigator deems it necessary on clinical grounds to give the IGIV during the period 32 to 90 days following the first treatment course, he/she, at his/her discretion, may give another 1 or 2 courses of GAMMAPLEX (treatment courses 2 and 3). See Figure 1 and Table 2.

If, however, it is considered that a second course of GAMMAPLEX would be appropriate after Day 9 and before Day 32, investigators must discuss this with the medical monitor and sponsor before proceeding. The administration of GAMMAPLEX during this period would interfere with the analysis of the duration of effect of GAMMAPLEX, an important secondary efficacy variable.

Before the first infusion of GAMMAPLEX in each additional treatment course, the following will be done (as detailed in Section 5.3.1.2):

Interval Medical History

Physical Examination

Vital Signs

Blood Samples for Laboratory Tests

The following samples will be taken before dosing on the first day of the treatment course only

- Platelet count (local laboratory)
- Blood for Laboratory B

Samples for Laboratory A will be collected at each infusion visit.

Direct Coombs' Test before the first infusion of each treatment course (see Section 9.3.2)

Reserve sample before the first infusion of each treatment course. (See Section 9.3.1)

Urinalysis before the first infusion of each treatment course

GAMMAPLEX Infusion

Each subject will be treated with GAMMAPLEX at a total dose of 2 g/kg body weight divided over 2 consecutive days, i.e., 1 g/kg on Day X and 1 g/kg on Day X+1 (see Sections 5.3.1.2 and 7.2). The date of additional treatment courses will be decided by the investigator based upon the subject's response to the first treatment course (for the second treatment course) and the second treatment course (for the third treatment course).

Adverse Events

Concomitant medications

5.3.1.5. Follow-Up Visits for Additional Treatment Course(s) (Infusions 3–6)

Subjects will return for a follow-up visit 3 days after the end of each of the second and third treatment courses, if they receive them (Day X+4 and Day Y+4 respectively). At those visits, the following will be done (see Section 5.3.1.2 and Table 2):

Interval Medical History

Physical Examination

Vital Signs

Blood Samples for Laboratory Tests

The following samples will be taken on Day X+4 and Day Y+4:

- Blood for Laboratory A

- Blood for Laboratory B

Urinalysis

Direct Coombs' Test

Samples for LDH, haptoglobin, and urine hemosiderin will also be taken to test for hemolysis (see Section 9.3.2)

Reserve Sample (see Section 9.3.1)

Adverse Events

Concomitant Medications

5.3.1.6. End of Study Visit

For all subjects this visit will be approximately 3 months after the first infusion of the last course of GAMMAPLEX and will be the End of Study visit for all subjects irrespective of whether they received 1, 2, or 3 courses. For subjects receiving 1 treatment course of GAMMAPLEX only, this visit will take place on Day 90.

Interval Medical History

An interval medical history will be performed. Any changes from the last medical history should be recorded in the CRF. Subjects should not receive any more GAMMAPLEX treatments after this visit.

Physical Examination

A final physical examination will be performed at this visit and the results recorded on the CRF.

Vital Signs

Vital signs assessed by qualified personnel will be entered in the CRF. **Before listing changes in vital signs as AEs, each change should be assessed for clinical relevance (see Section 9.2).**

Blood Samples for Laboratory Tests (see Section 9.3.1)

- Laboratory A
- Laboratory B

NAT for HCV, HIV, and Parvovirus B19

Serology for HIV1 and HIV2, HCV, and HBsAg

Serum for IgG, IgA, and IgM levels

Reserve Sample

Retention Sample (see Section 9.3.1)

Urinalysis

Adverse Events

All AEs reported by the subject or observed by the investigator will be recorded at each visit (see Sections 5.3.1.1 – 5.3.1.4 and 9.1.1).

Assessment for Bleeding/Hemorrhage

See Appendix 1.

Concomitant medications

See Sections 5.3.1.1 to 5.3.1.4.

Figure 1 Study Flow Chart

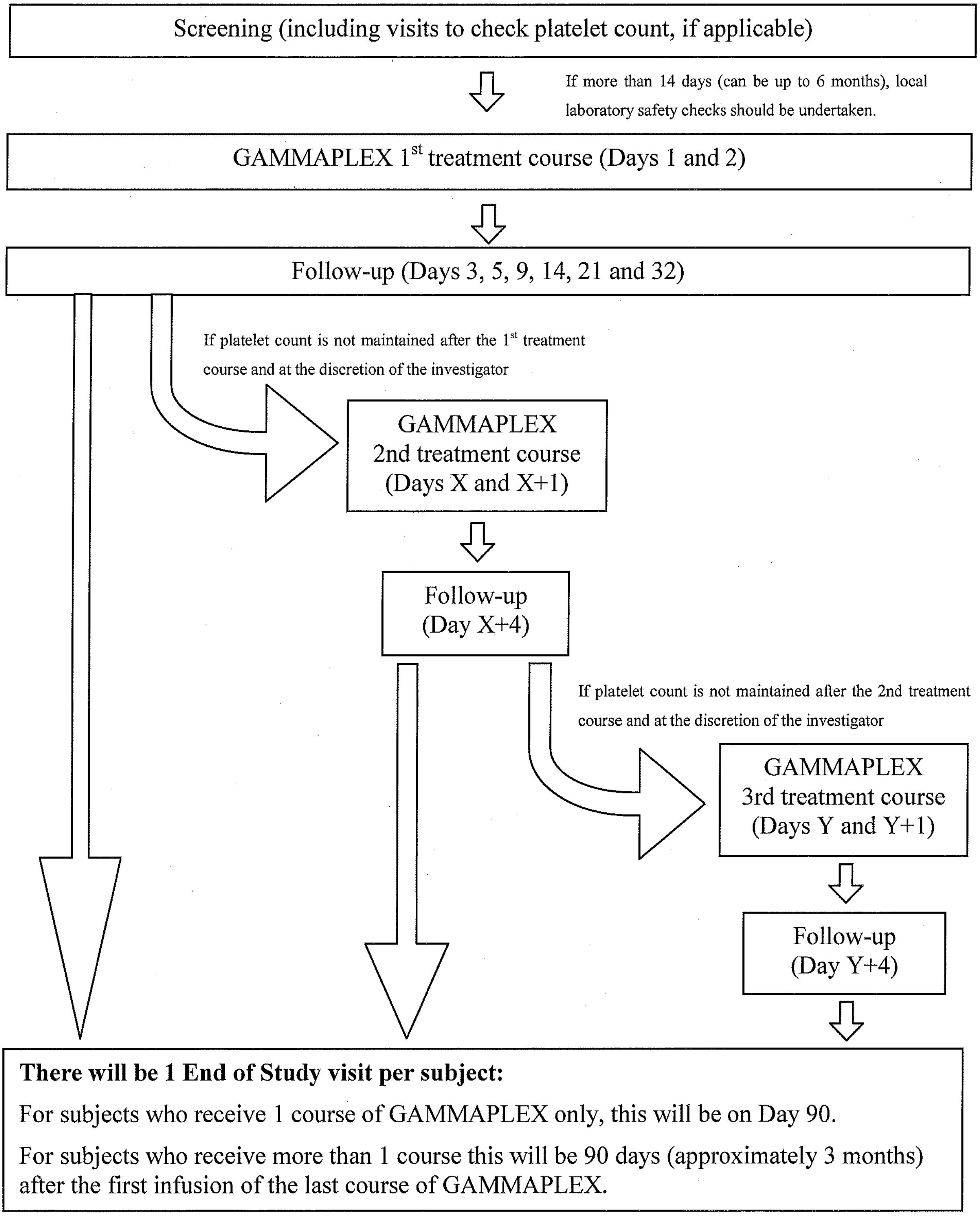

Table 1 Schedule of Assessments

Evaluation (Volume of Blood)	Visit name/Day/Time relative to first infusion on Day 1										
	Screening		Infusion 1	Infusion 2	Follow-up	Follow-up	Follow-up	Follow-up	Follow-up	Follow-up	End of Study visit (m)
	Full	Re-check (a)	Day 1	Day 2	Day 3 (±12 h)	Day 5 (±1 day)	Day 9 (±2 days)	Day 14 (±2 days)	Day 21 (±2 days)	Day 32 (±2 days)	Day 90 (±7 days)
Eligibility	X	X	X								
Consent	X										
HCG Urine Test (fertile females only)	X		X								
Complete Medical History	X										
Interval Medical History		X	X	X	X	X	X	X	X	X	X
Physical Examination	X	X	X	X	X	X	X	X	X	X	X
Vital Signs (b)	X	X	X	X	X	X	X				X
Local lab.–platelet count (2mL) (c)		X	X								
Local lab. – safety (7 mL) (d)		X									
Laboratory A (2 mL) (e)	X		X	X	X	X	X	X	X	X	X
Laboratory B (5 mL) (f)	X		X		X		X			X	X
Urinalysis	X		X		X		X			X	X
Direct Coombs' Test (2 mL) (g)	X		X			X (g)		X (g)			
Virology – NAT (13 mL) (h)	X		X				X (i)			X	X
Virology – Serology (8 mL) (j)	X		X							X	X
Immunoglobulins (5 mL) (k)	X		X	X	X	X	X	X	X	X	X
Reserve Sample (5 mL)	X		X	X	X	X	X	X	X	X	X
Retention Sample (2.5 mL)			X								X
Blood Typing (1 mL) (l)	X										
GAMMAPLEX infusion			X	X							
Adverse Events		X	X	X	X	X	X	X	X	X	X
Assessment for Bleeding/Hemorrhage	X	X	X	X	X	X	X	X	X	X	X
Concomitant Medications	X	X	X	X	X	X	X	X	X	X	X
Diary Cards			X	X	X	X	X	X	X	X	
**Total Blood Volume Required**	**40 mL**	**9 mL**	**44.5 mL**	**12 mL**	**17 mL**	**14 mL**	**21 mL**	**14 mL**	**12 mL**	**38 mL**	**40 mL**

a) For subjects screened more than 14 days prior to Day 1, a safety assessment should be done including taking blood samples for local laboratory measurement.

b) Vital Signs (blood pressure, heart rate, oral temperature, respiratory rate) should be taken as follows: 10 min before and at the start of each infusion, 10 min after initiation of each infusion, 10 min after each rate increase, 10 min and 30 min after maximum rate is achieved, every 60 minutes until infusion is complete, at the time the infusion stopped, and 15, and 30, after the infusion has stopped. Vital signs must be taken with the subject in the same position on each occasion.

c) On Day 1 (and visit(s) to check platelet count, if applicable) only, a platelet count will also be done locally prior to infusion to confirm eligibility of the subject (2mL).

d) For all subjects screened more than 14 days prior to Day 1, additional safety laboratory samples will be taken to be analyzed by the local laboratory e.g. CBC, LFTs, serum Creatinine/BUN.

e) Laboratory A includes CBC with differential and platelet count.

f) Laboratory B includes bilirubin, creatinine, BUN, ALT, AST.

g) Director Coombs' Test - on Days 5 and 14, samples for LDH, haptoglobin, and urine hemosiderin will also be taken.

h) NAT for HCV, HIV, and Parvovirus B19.

i) Parvovirus B19 only will be tested on Day 9; 4-mL blood sample.

j) Serology for HIV 1 & 2, HCV and HBsAg. **Note: If a positive result on an NAT or serologic test occurs, it will be repeated at the subject's next scheduled visit.**

k) Immunoglobulins - IgG (all visits) and IgA and IgM (Screening and at End of Study visit only).

l) The 1-mL sample needed for blood typing will come from the tube taken for the Direct Coombs' Test.

m) For all subjects this visit will be approximately 3 months after the last dose of GAMMAPLEX and will be the End of Study visit for all subjects irrespective of whether they received 1, 2, or 3 courses. For subjects receiving 1 treatment course of GAMMAPLEX only, this visit will take place on Day 90.

Table 2 Additional Assessments for Subjects who Require 2 or 3 Courses of GAMMAPLEX (Infusions 3 - 6)

**Evaluation (Volume of Blood)**	**Visit**						
	Treatment Course 2			Treatment Course 3			End of Study visit
	Infusion 3 (Day X)	Infusion 4 (Day X+1)	Follow-up (Day X + 4 ±1 day)	Infusion 5 (Day Y)	Infusion 6 (Day Y+1)	Follow-up (Day Y + 4 ±1 day)	90 days after the first infusion of the last course of GAMMAPLEX (± 7 days)
Interval Medical History	X	X	X	X	X	X	X
Physical Examination	X	X	X	X	X	X	X
Vital Signs (a)	X	X	X	X	X	X	X
Laboratory A (2 mL) (b)	X*	X	X	X*	X	X	X
Laboratory B (5 mL) (c)	X		X	X		X	X
Urinalysis	X		X	X		X	X
Direct Coombs' Test (2 mL) (d)	X		X (d)	X		X (d)	
Virology – NAT (13 mL) (e)							X
Virology – Serology (8 mL) (f)							X
Immunoglobulins (5 mL) (g)							X
Retention Sample (2.5 mL)							X
Reserve sample (5 mL)	X		X	X		X	X
GAMMAPLEX infusion	X	X		X	X		
Adverse Events	X	X	X	X	X	X	X
Assessment for Bleeding/Hemorrhage							X
Concomitant Medications	X	X	X	X	X	X	X
**Total Blood Volume Required**	**16 mL**	**2 mL**	**14 mL**	**14 mL**	**2 mL**	**14 mL**	**40.5 mL**

a) Vital Signs (blood pressure, heart rate, oral temperature, respiratory rate) should be taken 10 min before and at the start of each infusion, 10 min after initiation of each infusion, and 10 min after each rate increase, 10 min and 30 min after maximum rate is achieved, and every 60 minutes until infusion is complete, at the time the infusion stopped, and 15, 30 and 60 min after the infusion has stopped. Vital signs must be taken with the subject in the same position on each occasion.

b) Laboratory A includes CBC with differential and platelet count. ***Note: On Day 1 only, a platelet count will also be done locally prior to infusion to confirm eligibility of the subject (2mL).**

c) Laboratory B includes bilirubin, creatinine, BUN, ALT, AST.

d) Direct Coombs' Test - on Days X+4 and Y+4, samples for LDH, haptoglobin, and urine hemosiderin will also be taken.

e) NAT for HCV, HIV, and Parvovirus B19.

f) Serology for HIV 1 & 2, HCV and HBsAg. **Note: If a positive result on an NAT or serologic test occurs, it will be repeated at the subject's next scheduled visit.**

g) Immunology - IgG, IgA, and IgM on 3-month viral safety visit only.

6. STUDY POPULATION

6.1. Inclusion Criteria

1. Males and females aged between 18 and 70 years
2. Confirmed diagnosis of chronic ITP of at least 6 months duration.
3. Platelet count ≤20 x 10^9/L at enrollment (local laboratory).
4. Absence of other conditions that, in the opinion of the investigator, could cause thrombocytopenia.
5. If subjects are currently being treated with corticosteroids the treatment regimen/dose must have been stable (for a minimum of 2 weeks before Day 1). However, subjects must remain on a stable treatment regimen. If there is any intent to alter the corticosteroid treatment regimen (e.g., tapering of corticosteroids) before Day 10, subjects may not be included in the study. If possible, doses of corticosteroids or other immunosuppressant medications should remain constant until Day 32. The primary efficacy variable of the study is the platelet count at Day 9. Therefore, changing the subject's medication regimen should be avoided in order to reduce the variables that could have an effect on the platelet count.
6. If subjects are currently being treated with cyclophosphamide, azathioprine or attenuated androgens, the treatment regimen and dose must have been stable for a minimum of 2 months before Day 1. However, if there is any intent to alter the treatment regimen before Day 10, subjects may not be included in the study. If possible, doses of corticosteroids or other immunosuppressant medications should remain constant until Day 32. The primary efficacy variable of the study is the platelet count at Day 9. Therefore, changing the subject's medication regimen should be avoided in order to reduce the variables that could have an effect on the platelet count.
7. Splenectomized subjects and both Rh(D)+ and Rh(D)- subjects can be included.
8. The subject has signed an informed consent form (subjects must be at least 18 years old), and/or the subject's legal guardian has signed the informed consent form if indicated, not more than 6 months prior to Day 1 (see Section 11.3).
9. If a subject is a female of child-bearing potential, she must have a negative result on a urine-based HCG pregnancy test.
10. If a subject is a female who is or becomes sexually active, she must practice contraception by using a method of proven reliability for the duration of the study.

6.2. Exclusion Criteria

1. The subject has a history of any severe or anaphylactic reaction to blood or any blood-derived product, or any severe reaction to IGIV or any other IgG preparation.
2. The subject is known to be intolerant to any component of the investigational product.

3. The subject has received any live virus vaccine within the last 3 months.
4. The subject has received an IGIV preparation within 1 month prior to Day 1.
5. The subject is currently receiving, or has received, any investigational agent within the 1 month prior to Day 1.
6. The subject has received any blood, blood product, or blood derivative within the 1 month prior to Day 1.
7. The subject has received Rituximab within the 3 months before Day 1.
8. The subject is pregnant or is nursing.
9. The subject is positive for any of the following at screening:
    - HBsAg
    - NAT for HCV
    - NAT for HIV
    - Antibodies to HCV or HIV 1 or 2
10. The subject, at screening, has levels greater than 2.5 times the upper limit of normal, as defined by the central laboratory, of any of the following:
    - Alanine aminotransferase (ALT)
    - Aspartate aminotransferase (AST)
11. The subject has a severe renal impairment (defined as serum creatinine greater than 2 times the upper limit of normal or BUN greater than 2.5 times the upper limit of normal for the range of the laboratory doing the analysis); the subject is on dialysis; the subject has a history of acute renal failure.
12. The subject is known to have abused alcohol, opiates, psychotropic agents, or other chemicals or drugs within the past 12 months.
13. The subject has a history of deep vein thrombosis (DVT) or thrombotic complications of IGIV therapy
14. The subject has any history or sign of hyperviscosity, transient ischemic attack (TIA), stroke, other thromboembolic event, or unstable angina
15. The subject suffers from any acute or chronic medical conditions (e.g., renal disease or predisposing conditions for renal disease, coronary artery disease, or protein losing enteropathy) that, in the opinion of the investigator, may interfere with the conduct of the study
16. The subject has an acquired medical condition, such as chronic lymphocytic leukemia, lymphoma, multiple myeloma, chronic or recurrent neutropenia (defined as an absolute neutrophil count (ANC) $< 1 \times 10^9$/L).
17. The subject has non-controlled arterial hypertension (systolic blood pressure >160 mmHg and/or diastolic blood pressure >90 mmHg).

18. The subject is anemic (hemoglobin <10 g/dL) at screening.
19. The subject is unlikely to adhere to the protocol requirements of the study or is likely to be uncooperative.

6.3. Subject Withdrawal

Subjects may be withdrawn from study for any of the following reasons:

1. The subject withdraws from the study on his/her own volition.
2. The subject develops Hepatitis B, Hepatitis C, or HIV as determined by a positive result on a test for HBsAg or on a NAT for HCV or HIV RNA, or the appropriate serological tests for these viruses.
3. The subject has a serious adverse event (SAE) while participating in the study and, at the discretion of the site investigator, is discontinued.
4. The subject becomes pregnant while participating in the study. Any subject that becomes pregnant while participating in the study will not receive any further infusions of GAMMAPLEX.
5. The investigator may withdraw subjects at anytime if they feel it is not in the subject's best interest to continue in the study.

If a subject's Day 1 blood sample results from the central laboratory (for Laboratory A, Laboratory B tests or for the other protocol required blood tests) are out of range, the subject will be withdrawn from the efficacy analysis only. These subjects will still be included in the safety analysis and will therefore continue to attend the follow-up visits. Subjects withdrawn from the efficacy analysis will not be eligible to receive any further courses of GAMMAPLEX.

If a subject receives any of the study product and withdraws, all of the assessments scheduled on Day 32 and the End of Study visit must be done, if possible. For subjects who received more than 1 course of GAMMAPLEX before withdrawal, every effort must be made to complete the assessments scheduled for the End of Study visit.

6.4. Study Termination

In the event of a trend in the safety data that would indicate any concern for the safety of the subjects, the sponsor, medical monitor and the study site investigator will consider if the study should be terminated. The appropriate IRBs/IECs and regulatory authorities will be informed of any decision to terminate the study earlier than planned.

7. DESCRIPTION OF TREATMENT ADMINISTERED

7.1. Investigational Product

7.1.1. Identification and Batch Numbers

The batch numbers of GAMMAPLEX will be documented in the Certificate of Analysis that will be filed in the Trial Master File. The actual batch number will be documented in the CRF for

each infusion. A list of all batches used and the subjects who received them will be included in the final study report. At least 2 batches of GAMMAPLEX will be used for this study.

7.1.2. Packaging and Labeling

GAMMAPLEX will be manufactured, packed, and labeled by BPL. The product will be supplied in glass bottles (100 mL per bottle), and each bottle will be packed in a box. The label will include contact information for BPL and the identity, total quantity and volume, concentration, batch number, expiry date, and storage requirements of the product. A statement (in bold type) that the investigational product is not compatible with other fluids will also be on the label. The label on the bottle will be a 2-part, tear-off label. One part must be attached to the subject's source documents when the bottle is used for the infusion.

7.1.3. Conditions for Storage and Use

GAMMAPLEX should be stored in a secure area of limited access, preferably in a locked cupboard, protected from light, and maintained at a temperature of 36°F to 77°F (2°C to 25°C). GAMMAPLEX must not be frozen.

7.2. Investigational Treatment

7.2.1. Dose Schedule and Rationale

All eligible subjects will receive an infusion of 1 g/kg of GAMMAPLEX on each of 2 consecutive days (Day 1 and Day 2). This will be the first treatment course of GAMMAPLEX. The dose of 1 g/kg/day on 2 consecutive days is consistent with recommendations for the use of other IGIV products for ITP [15-17,21].

A small variation in the amount of GAMMAPLEX to be infused will be allowed for those subjects whose calculated dose would necessitate disposing of a significant amount of GAMMMAPLEX following the infusion. In order to avoid unnecessary waste, the total volume of GAMMAPLEX infused may be adjusted by the lesser of either 10 mL or 2% of the total volume infused.

If the platelet count is not maintained for the desired length of time after the first course of GAMMAPLEX, and at the discretion of the study investigator and subject/guardian, subjects may receive up to 2 further courses of GAMMAPLEX at the same dosage regimen as course 1 between Day 32 and the End of Study visit.

7.2.2. Product Preparation

Before use, all bottles must be inspected visually for discoloration, particles, and fibers; if the contents of a bottle are cloudy or contain particulate material, they must not be used. GAMMAPLEX is to be at room temperature when it is infused.

One of two methods of administration may be used:

From the bottle

Administration of GAMMAPLEX must be started immediately after breaking the seal of the bottle. A qualified member of staff must administer GAMMAPLEX by using a standard administration kit. **For any single treatment course, all GAMMAPLEX given to any 1 subject must be from the same batch.**

From an infusion bag

GAMMAPLEX must be pooled under aseptic conditions into a polyvinyl chloride infusion bag by a qualified member of the site staff or pharmacist working to local standard operating procedures (SOPs). The infusion must be started as soon as possible, and in any case within 2 hours, after pooling into the infusion bag.

All infusions will be administered directly from the infusion bag by using a standard administration kit. The infusion bags will be labeled with the manufacturer's lot/batch number, subject number, protocol number, and the expiration date. **For any single treatment course, all GAMMAPLEX given to any 1 subject must be from the same batch.**

7.2.3. Method of Administration

All subjects should be adequately hydrated before infusion. If clinical assessment suggests the subject is dehydrated, intravenous fluids may be given before the scheduled infusion is started. Infusions will be administered by trained personnel under the direct supervision of the responsible investigator or his/her designee.

GAMMAPLEX will be administered intravenously (peripheral or central vein) through an infusion line with a 15- to 20-micron filter. Mixing and administration of any other product (including normal saline) with GAMMAPLEX is strictly prohibited.

All infusions will be initiated at a rate of 0.01 mL/kg/min for the first 15 minutes; if tolerated, the rate of infusion must be advanced as shown in Table 3. Lack of tolerance at any of these rates must be recorded as an AE at that rate. If a subject has the same AE at the same rate twice, and those AEs have been recorded, then subsequent infusion rate escalations should be halted at the previously highest tolerated rate.

Table 3 Starting, Incremental, and Maximum Infusion Rates

Infusion	GAMMAPLEX Infusion Rate (mL/kg/min)	(mg/kg/hr)	Elapsed Time (min)
Starting	0.01	30	0–15
Increment to	0.02	60	16–30
	0.04	120	31–45
	0.06	180	46–60
Maximum	0.08	240	61 until end of infusion

If any AE of moderate or severe intensity occurs (see Table 4), the rate of the infusion at which this AE occurred will be recorded, and the infusion rate will be reduced to one-half the rate of that at which the event was observed; or if necessary, to a keep open (TKO) rate until symptoms subside. The infusion may then be resumed at a rate tolerated by the subject.

7.2.4. Product Accountability

An Investigational Product Accountability Record for GAMMAPLEX must be kept current by the clinical site and must contain:

- subject number and initials
- initials of the staff person dispensing the product
- dates and quantities of investigational product received with batch numbers
- dates and quantities of investigational product dispensed per subject with batch numbers
- dates and quantities of investigational product returned per subject with batch numbers

The Investigational Product Accountability Record must be completed to document receipt, dispensing, and running total of product at site and must be included in the shipment of all unused GAMMAPLEX to the central distributor. At the end of the study, the site must prepare a final Investigational Product Reconciliation Statement; this form will be collected by the monitor. These forms must be made available throughout the study for inspection by authorized representatives of the CRO, manufacturer/sponsor, and regulatory agencies. The investigator is responsible for the accountability of all used and unused product and supplies used in the study. Where available, affix the tear-off portion of the label to the source documentation or record in writing the batch number used for the infusion.

Any empty or partly used bottles of GAMMAPLEX must be retained at site until instruction is given that they may be disposed of. This will be after the monitor has inspected the accountability records. The monitor will then instruct site staff that bottles may be destroyed at site. A certificate of destruction must be completed to document this.

GAMMAPLEX must not be used outside of this protocol and must not be used for subjects who are not in this study.

7.3. Treatment Compliance

GAMMAPLEX will be administered intravenously and only under medical supervision; therefore, compliance is not an issue in this study.

8. ASSESSMENT OF EFFICACY

8.1. Primary Efficacy Variable

The primary efficacy variable will be the percent of subjects attaining a platelet count of $\geq 50 \times 10^9/L$ by Day 9 (the 7th day after completing the second infusion).

8.2. Secondary Efficacy Variables

There will be 2 secondary efficacy variables:

- Duration of time for which the platelet count remains $\geq 50 \times 10^9/L$ following the first treatment course of GAMMAPLEX
- Changes in the signs of any bleeding/hemorrhage up to Day 32 (see Appendix 1 for grading criteria)

9. ASSESSMENT OF SAFETY

9.1. Safety Variables

The variables used to assess safety will be the following:

- Adverse events
  - The number and percent of infusions with at least 1 AE that occurs during an infusion or within 72 hours after the infusion stops
  - Nature, severity, and frequency of AEs
  - Suspected unexpected serious adverse reactions (SUSARs)
- Vital signs
- Clinical laboratory tests and Direct Coombs' Test
- Transmission of viruses
- Physical examination

9.1.1. Adverse Events

For subjects who receive only 1 treatment course of GAMMAPLEX on Days 1 and 2, AEs will be documented from the date the informed consent form is signed until the End of Study visit on Day 90. For subjects who receive (an) additional course(s) of GAMMAPLEX, AEs will be documented from the date the informed consent form is signed until the End of Study visit which for this group of subjects is 90 days, approximately 3 months, after the first infusion of the last course of GAMMAPLEX. AEs will be reviewed and documented at each visit by examining the diary cards (for the first course of GAMMAPLEX up to Day 32) and by direct questioning of the subject.

The investigator will attempt to establish a diagnosis of the AE based on signs, symptoms, and/or other clinical information. It is the diagnosis that should be documented as the AE and not the individual symptoms.

9.1.1.1. Definitions

Adverse Event

An AE is any untoward medical occurrence not necessarily having a causal relationship to treatment. An AE can, therefore, be any unfavorable and unintended sign (including an abnormal laboratory finding), symptom, or disease (new or exacerbated) temporally associated with the use of a medicinal product, whether or not considered related to the medicinal product.

Unexpected Adverse Event

An AE is defined as unexpected if it and its severity are not indicated in the current Investigator's Brochure.

Infusion-Related Adverse Events

All AEs that occur from the start of the infusion until 72 hours after the infusion will be recorded as infusion-related, but will not necessarily be related to GAMMAPLEX per se.

Clinical Laboratory and Other Adverse Events

If a laboratory result is considered by the investigator to be clinically significant or have a clinically significant pathological change from baseline, it should be recorded as an AE.

Other abnormal results (e.g., x-rays, scans, physical examination findings) that worsen from baseline and/or are considered clinically significant should also be recorded as AEs.

The investigator will exercise his or her medical and scientific judgment in deciding whether an abnormal laboratory finding or other abnormal assessment is clinically significant.

Serious Adverse Event

An SAE is any untoward medical occurrence that, at any dose:

- Results in death
- Is life-threatening (places the subject at <u>immediate</u> risk of death)
- Requires inpatient hospitalization or prolongs existing hospitalization (does not include hospitalizations for elective procedures for pre-existing conditions that did not worsen from baseline)
- Results in persistent or significant disability/incapacity
- Is a congenital anomaly/birth defect
- Is an important medical event (event that may not be immediately life-threatening or result in death or hospitalization but may jeopardize the subject or may require medical or surgical intervention to prevent one of the other outcomes listed in the

above definition. Examples include: allergic bronchospasm requiring intensive treatment, blood dyscrasias or convulsions, and the development of drug dependency or drug abuse)

Suspected Unexpected Serious Adverse Events

All AEs judged to be related to GAMMAPLEX that are both unexpected and serious (SUSARs) are subject to expedited reporting in the U.K. BPL will be responsible for reporting these to the concerned competent authorities.

9.1.1.2. Assessment of Severity

The investigator will make an assessment of severity for each AE and SAE reported during the study. Severity refers to the grading of adverse events to assess the severity of symptoms as evaluated by the investigator or as experienced by the subject. The severity grading is independent of seriousness; in other words, a severe event is not necessarily serious.

AEs Occurring During the Infusion and up to 60 minutes from the End of Infusion

The severity of AEs that occur during or less than 60 min after an infusion will be graded according to the criteria in Table 4.

AEs Occurring Later than 60 minutes After the End of Infusion

The severity of AEs that occur more than 60 min after the end of an infusion will be graded as follows:

- **Mild**: Event resolved spontaneously or no treatment was necessary beyond administration of non-prescription drugs.
- **Moderate**: Event required use of prescription drugs and produced no sequelae.
- **Severe**: Event produced sequelae or required a visit to health care professional.

Table 4 Criteria for Grading Severity of Infusion-Related Adverse Events[a] that Occur During Infusion or up to 60 minutes from the End of Infusion

TYPE OF EVENT Sign/Symptom	Mild	Moderate	Severe
**CARDIOVASCULAR**			
Tachycardia	HR = 1.2 to 1.4x baseline	HR = 1.41 to 1.6x baseline	HR > 1.6 x baseline
Arrhythmia	Occasional, asymptomatic (not-present pre-infusion)	< 1 per minute, and asymptomatic	> 1 per minute, or symptomatic
Hypotension (% fall in systolic or diastolic blood pressure in mmHg (from either pre-infusion measurement, whichever is lower – see Section 9.2.	10 to 15%[b]	16 to 25%[b]	> 25%[b]
Hypertension (% rise in systolic or diastolic blood pressure in mmHg (from either pre-infusion measurement, whichever is higher – see Section 9.2.	20 to 25%[b]	26 to 30%[b]	> 30%[b]
**ALLERGIC**	Pruritus without rash	Pruritic rash; tachypnea 1.3 to 2 x the higher baseline, chest tightness suggestive of bronchospasm	Urticaria; tachypnea > 2 x the higher baseline, bronchospasm, laryngospasm, angioedema, circulatory shock (severe hypotension)
**SYSTEMIC**			
Fever °F (°C)	99 to 101.2 (37.7 to 38.4)	101.3 to 104 (38.5 to 40.0)	>104 (40)
Chills	Feels cold, slight shivering	Intermittent shaking	Continuous shaking, cold and clammy skin
Headache	Discomfort, but does not require medication	Responds to non-narcotic analgesia	Requires narcotic analgesia or persistently recurrent despite treatment
Other pain	Discomfort, but does not require medication	Pain requiring non-prescription medication.	Pain requiring prescription and/or narcotic medication
Nausea	Discomfort, but does not require medication	Can take fluids without antiemetic medication	Little or no oral intake and/or requires antiemetic medication
Vomiting	1 episode	2 to 3 episodes	> 3 episodes

[a] This table applies to AEs associated with an infusion that occur during an infusion and/or up to 60 minutes after an infusion. The infusion rate at which the AE is observed will be recorded.

[b] If not accompanied by an increase in heart rate and subject is assessed as being comfortable or sleeping, blood pressure changes of this magnitude may be classified by the investigator as physiologic and not considered an AE.

9.1.1.3. Assessment of Relationship

The investigator must provide his/her opinion of the relationship between GAMMAPLEX and the occurrence of each AE. The relationship of each AE will be categorized as follows:

- Not Related: Event for which sufficient evidence exists to conclude that the etiology is unrelated to the study product.
- Possibly Related: There is some temporal relationship between the event and the administration of the study product, and the event is unlikely to be explained by the subject's medical condition or other therapies.
- Probably Related: The temporal relationship between the event and administration of the study product is suggestive, and the event is less likely to be explained by the subject's medical condition or other therapies.
- Definitely Related: The event follows a reasonable temporal sequence from administration of the study product, follows a known or suspected response pattern to the product, is confirmed by improvement upon stopping the product (dechallenge) and reappears upon repeated exposure (rechallenge), if that occurs.

If an AE occurs that could equally be caused by GAMMAPLEX or other factors, the AE must be recorded as either Probably or Possibly related to GAMMAPLEX.

There may be situations when an SAE has occurred and the investigator has minimal information to include in the initial report. However, it is very important for the investigator to make an assessment of relationship for every event before transmission of the SAE form since it is used in determining expedited reporting requirements of the case to regulatory authorities. The investigator may change his/her opinion of causality as additional information becomes available and amend the SAE forms as necessary.

9.1.1.4. Follow-Up of Adverse Events

All AEs will be followed until resolution, until the condition stabilizes, or until the subject is lost to follow-up. The investigator will ensure that follow-up includes any supplemental investigations that may be indicated to determine the nature and/or causality of the AE or SAE. The sponsor may require that the investigator perform or arrange for the conduct of additional investigations (not otherwise required per protocol) to determine the nature and/or causality of the AE or SAE.

Any subject that becomes pregnant during the study must be followed up to delivery.

9.1.2. Submission of Serious Adverse Event Reports

When an SAE occurs, it is the responsibility of the investigator to review all documentation (e.g., hospital progress notes, diagnostic reports, laboratory assessments) relative to the event. The investigator will then be responsible for recording all relevant information on the appropriate AE CRF and SAE form. New information or corrected information will be recorded on a follow-up SAE report.

Any SAE that occurs at any time between the signing of the informed consent and 30 days after the last dose of study medication, regardless of the relationship to study medication, must be reported on the SAE CRF, and a copy of this form must be faxed to the drug safety manager, and in Argentina to ACTIVA-CRO, at the fax number(s) listed below within 24 hours of knowledge of the event:

Manager, Drug Safety

INC Research Inc.

Fax: (U.S. country code) 877-464-7787

ACTIVA-CRO

Fax: 011-5290-8273

The INC Research SAE Hotline must be called at the same time the fax is sent.

INC Research SAE Hotline

(U.S. country code) 877-462-0134

Please contact the person listed above if there are questions regarding completion of the SAE CRF.

9.1.3. Regulatory Reporting Requirements for Serious Adverse Events

The sponsor has a legal responsibility to notify, as appropriate, regulatory authorities about the safety of a product under clinical investigation. Prompt reporting of SAEs by the investigator to the drug safety manager is essential so that legal and ethical requirements for the safety of other subjects are met. The drug safety manager will be responsible for reporting all SAEs to the sponsor and to the FDA; the sponsor will be responsible for reporting SAEs to the European regulatory authorities. The sponsor and/or designee (e.g., CRO) will report SAEs to other pertinent regulatory authorities (i.e., in India and Argentina) as indicated in the figure below.

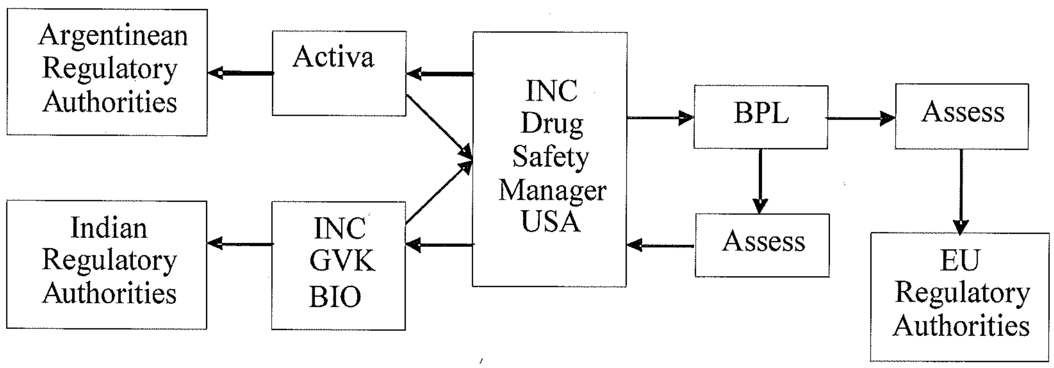

Details of the reporting procedures for SAEs are outlined in the SAE Management Plan designed for this study by the CRO.

The investigator will comply with local requirements related to the reporting of SAEs, safety notifications, and other safety information related to the trial to their local IRB/IEC. Reporting of safety information will be according to ICH/GCP guidelines and will follow requirements of the countries in which study sites are located.

9.1.3.1. Regulatory Reporting Requirements for SUSARS

All elements required by the European Union for reporting suspected unexpected serious adverse reactions (SUSARs) are in the Study Manual. Reporting will be according to ICH/GCP guidelines and will follow requirements of countries in which study sites are located.

9.2. Vital Signs

Vital sign values will be obtained as detailed in Section 5.3.1.2. Any clinically significant changes in vital signs will be reported as AEs as described in Section 9.1.1.2. Clinically significant hypotension (i.e., a $\geq$ 30 mmHg drop in systolic blood pressure accompanied by clinical findings/symptoms, or a systolic blood pressure of $\leq$ 70 mmHg) and/or anaphylactic reactions (significant hypotension with generalized hives/angioedema or bronchospasm) warrant discontinuation of the infusion and administration of appropriate supportive care as deemed necessary by the investigator (may include epinephrine, antihistamine, corticosteroids, supplemental oxygen, expansion of the plasma volume as necessary, etc.). These occurrences must be recorded in the CRF as AEs. At the discretion of the investigator, the subject may be withdrawn from the study, but will complete the follow-up visits. Before listing changes in vital signs as AEs, each change should be assessed for clinical relevance.

9.3. Clinical Laboratory Tests and Direct Coombs' Test

9.3.1. Clinical Laboratory Tests

Blood should be drawn according to the procedures outlined in the Laboratory Manual provided to each investigative site by the central laboratory. The volume of blood for each sample per study visit is in Table 1.

On Day 1 of each infusion and visit(s) to check platelet count (if applicable), the subject's platelet count will also be done locally.

Additionally, for those subjects screened more than 14 days prior to Day 1 of the first infusion, a blood sample for assessment by the local laboratory must be taken to assess CBC, LFTs, serum Creatinine/BUN prior to enrolment in the study.

Blood samples will be drawn at scheduled times for the following laboratory determinations:

Laboratory A:	Laboratory B:
• CBC with differential<br>o WBC<br>o RBC<br>o Hematocrit<br>o Hemoglobin<br>o Platelet Count<br>o Neutrophil %<br>o Lymphocyte %<br>o Eosinophil %<br>o Monocyte %<br>o Absolute Neutrophil<br>o Absolute Lymphocyte<br>o Absolute Eosinophil<br>o Absolute Basophil<br>o Absolute Monocyte<br>• Platelet count	• Bilirubin<br>• Creatinine<br>• BUN<br>• ALT<br>• AST

In addition, the following will be done:

- NAT for HCV, HIV and Parvovirus B19
- Serology for HIV_1 and HIV_2, HCV, HBsAg.

If a positive result on an NAT or serologic test occurs, it will be repeated at the subject's next scheduled visit.

- IgG, IgA and IgM will be monitored at the End of Study visit and compared with the values obtained at screening. In addition, IgG will be monitored pre-infusion on Days 1 and 2 and at the study visits on Days 3, 5, 9, 14, 21 and 32, for the first course of GAMMAPLEX only.
- Reserve sample. A 5-mL blood sample (minimum 1 mL serum) will be obtained on each occasion that Laboratory A samples are taken and will be stored at -70°C at the central laboratory in the event that repeat tests are required. These samples will be discarded at completion of the study.
- Retention sample. In order to comply with the European CPMP requirements [22], a 2.5-mL blood sample will be collected from each subject immediately before the first infusion and at the End of Study visit. Serum (1 mL) will be stored in 2-mL tubes at -70°C for 15 years. These serum samples will be stored at a central archive (Cryo-Store).

Samples will be used for serology and NAT testing if required in the future. The central laboratory will coordinate the handling and shipping of these samples.

- Urinalysis

9.3.1.1. Effects on Hepatic, Renal, and Hematologic Function

If the results of any blood test demonstrate a rise to 2.5 times the upper limit of normal in AST, ALT, or bilirubin, these laboratory results will be documented as an AE. An additional blood sample will be drawn from the subject, and the tests will be repeated. The samples will also be tested serologically for HBsAg, and HCV, and HIV will be tested by NAT within a 1-week period.

9.3.2. Direct Coombs' Test

If any Direct Coombs' Test is positive, the central laboratory must notify the site investigator and the CRO of the results of all the above tests within 2 business days.

9.4. Viral Transmission

If any of the tests for HBV, HCV, HIV, or Parvovirus B19 suggest a change in viral status of the subject, the tests will be repeated by the central laboratory as soon as possible. If a change in viral status is confirmed by a second independent test, the reserve sample taken before the first infusion of GAMMAPLEX will be re-tested in the same laboratory. The central laboratory will inform the sponsor and the CRO within 12 hours of the change in viral status.

9.5. Physical Examination

Physical examination will be performed by personnel qualified to perform such procedures in the state in which the study site is located. Elements of the exam are as follows:

- General appearance
- Head, eyes, ears, nose, throat
- Chest
- Cardiovascular
- Respiratory
- Abdomen
- Musculoskeletal
- Genitourinary
- Skin
- Assessment of bleeding/hemorrhage (see Appendix 1)
- Other

Any clinically significant change from baseline in any system will be recorded as an AE.

10. STATISTICAL METHODS

10.1. Sample Size

The calculation of sample size was based on the primary efficacy variable, i.e., the percent of subjects attaining a platelet count of ≥50 x 10^9/L by Day 9 on the first course of GAMMAPLEX. A minimum of 31 evaluable subjects will be enrolled in the study. However, when 27 evaluable subjects have completed Day 9, a partial analysis may be conducted, and the response rate (P_1) calculated and compared with that of the historical control, P_0 (lower bound of 95% confidence interval) is greater than 0.60, to determine the power achieved at this interim stage. The response rate estimated at this stage will be used to re-estimate the sample size necessary at the end of the study to achieve a power of 80% to detect the desired difference.

10.2. Analysis Population

Any subject who started treatment with GAMMAPLEX and whose central laboratory results taken prior to infusion on Day 1 are within specified protocol parameters, will be included in the intent-to-treat (ITT) population for analysis of efficacy and safety. Any subject whose central laboratory results from Day 1 are outside of the required parameters, will be included in the safety analysis only.

Additionally, subjects aged 6 to 17 years (applicable only to subjects enrolled before implementation of Protocol Version 7) will be analyzed separately from the remaining samples for any differences in response to treatment. This subset will be analyzed in the same manner as the total sample.

10.3. Efficacy Analyses

10.3.1. Analysis of the Primary Efficacy Variable

The primary efficacy data will be analyzed by using the ITT.

The percent of subjects attaining a platelet count of ≥50 x 10^9/L by Day 9 will be compared with the historical response rate of >60%. A 1-sided 95% confidence interval will be constructed for the percent of subjects attaining a platelet count of ≥50 x 10^9/L by Day 9 for GAMMAPLEX. If the lower bound of this confidence interval is >0.60, GAMMAPLEX will be declared effective for the treatment of chronic ITP.

10.3.2. Analysis of the Secondary Efficacy Variables

- The method of Kaplan and Meier will be used to produce plots of the distribution of the time for which the platelet counts remain ≥50 x 10^9/L. A 95% confidence interval for the median time will be constructed.
- Each bleeding/hemorrhage event will be graded according to the criteria in Appendix 1 and analyzed by using descriptive statistics. Data will be summarized by number and percent.

10.4. Safety Analyses

10.4.1. Adverse Events

The number of infusions in which an AE occurs during an infusion or within 72 hours after the infusion stops will be tabulated. A summary table including the following will be produced:

- number of subjects with AEs
- number of subjects with SAEs
- number of subjects who died
- number of subjects who withdrew because of AEs
- number of subjects with product-related AEs.

The nature, severity and frequency of all AEs will be listed.

10.4.2. Other Safety Assessments

Summary tables for the results of laboratory measures of hepatic, renal, and hematologic function will include the following:

- Descriptive statistics (mean, standard deviation, median, maximum and minimum) for original values and change from baseline for all continuous variables
- Incidence (number and percent) of clinically significant, treatment-emergent abnormal values (TEAVs)
- Shift tables showing the change from baseline in the incidence of new or worsening clinically significant findings.
- The number of subjects in whom there is a transmission of Hepatitis B, Hepatitis C, HIV, or Parvovirus B19 will be tabulated. The results from the Direct Coombs' Test and associated haptoglobin, LDH and urine hemosiderin will also be tabulated.

All abnormalities after baseline in the results of the physical examination will be summarized by using number and percent. Shift tables from normal to abnormal and abnormal to normal will also be produced.

Summary statistics (mean, median, standard deviation, maximum and minimum of original values, and change from baseline) for all vital sign measures, will be provided.

10.5. Interim/Partial Analyses

There may be an interim analysis and a partial analysis performed during the study.

10.5.1. Interim Analysis

An interim analysis may be performed when at least 30 evaluable adult subjects have completed Day 32. All other subjects recruited up to this time point, e.g. non-evaluable subjects or children (applicable to subjects enrolled before implementation of Protocol Version 7) will be analyzed for safety only. The purpose of this analysis is to facilitate submission of an application to the U.K. and EU regulatory authorities (22).

10.5.2. Partial Analysis

A partial analysis may be performed when at least 27 evaluable subjects have completed Day 9. The observed response rate will be estimated and compared with the historical control of >0.60 to re-estimate the sample size.

11. ETHICAL AND REGULATORY COMPLIANCE

11.1. Ethical / Regulatory Framework

This study will be conducted in accordance with the ethical principles that have their origins in the Declaration of Helsinki. The study will be conducted in compliance with the protocol, ICH GCP regulations, and all applicable regulatory requirements. The clinical trial will be conducted in accordance with FDA 21CFR 50, Subpart B-Informed Consent of Human Subjects and the applicable regulatory requirements.

11.2. Institutional Review Board

The study protocol, subject information, informed consent form, and any other materials provided to the subjects will be submitted by the investigators to their IRB/IEC. The letter from the IRB/IEC approving the study must be available before any subject is exposed to a study-related procedure.

11.3. Subject Information and Informed Consent

The investigator will obtain written informed consent from each subject participating in this study, after adequate explanation of the aims, methods, anticipated benefits, and potential hazards of the study and before applying any study-related procedure to a subject. In Argentina this procedure should be witnessed and the witness must sign the consent form to document that the procedure has been followed.

For subjects not qualified to give legal consent, written consent must be obtained from the subject's legal guardian. In Argentina, minors (e.g., between 18 and 20 years of age) also require the signature of both parents (mother and father) or the legal guardian. In addition, the subject/subject's legal guardian (as applicable) will sign an appropriate form authorizing release of the subject's HIV test results to appropriate authorities as indicated. Publishing results of HIV subjects to authorities will not be allowed from Indian Local Ethics Committees. All consent forms must be approved in advance by the IRB/IEC (and the Ministry of Health in Argentina).

If a subject is unable to read or if their legal guardian is unable to read, an impartial witness should be present during the entire informed consent discussion. After the informed consent form and any other written information is provided, read, and explained to the subject or their legal guardian, and after oral consent has been obtained, if capable of doing so, the subject or their guardian should sign and date the informed consent form. The witness should also sign and date the consent form. In addition, the subject or their guardian should, if possible, sign an additional form confirming that the materials provided have been read and explained to them. The witness should also sign and date this additional form.

The investigator will complete and sign the informed consent section of the CRF for each subject enrolled.

12. QUALITY CONTROL AND QUALITY ASSURANCE

12.1. Source Data and Records

Source data are defined as all information related to clinical findings, observations, or other activities in the study, written in original records or consisting of certified copies of original records. The investigator will permit trial-related monitoring, audit(s), IRB/IEC review(s) and regulatory inspection(s), by providing direct access to source data/records.

12.2. Periodic Monitoring

The study monitor will contact and visit the investigator regularly and will be allowed, on request, to inspect the various records of the study. It will be the monitor's responsibility to inspect the CRFs at regular intervals throughout the study, to verify the adherence to the protocol and the completeness, correctness and accuracy of all CRF entries. The study monitor should have access to laboratory test reports and any other source records and data needed to verify the entries on the CRFs. The investigator agrees to co-operate with the study monitor to ensure that any problems detected in the course of these monitoring visits are resolved.

A "Delegation of Authority Log" will be filled in and signed by the responsible investigator. In accordance with this authority log, study site staff (e.g., co-investigators and nurses) will be authorized to enter data into the CRF. The monitor will visit the study centers at least every 6-weeks, any periods longer than this to be agreed with the investigator and sponsor.

12.3. Audit and Inspection

The investigator will make source data and documents for this trial available to a qualified clinical quality assurance auditor as mandated by sponsor and/or to health authority inspectors, after appropriate notification. The main purposes of an audit or inspection are to confirm that the rights and welfare of the subjects have been adequately protected, and that all data relevant for the assessment of safety and effectiveness of the investigational product have been reported to the sponsor. The verification of the CRF data may be performed by direct inspection of source documents, after the investigator has protected all confidential personal subject information not related to the study, in accord with local regulations.

12.4. Confidentiality of Subjects' Data

The investigator will ensure that the subject's anonymity is preserved.

13. DATA HANDLING AND RECORD KEEPING

13.1. Case Report Forms

For each subject enrolled, CRFs will be completed in English and signed by the investigator or an authorized co-investigator. All paper source documents will be filled out using an indelible pen with black ink, and must be legible.

13.1.1. Changes to Case Report Form Data

For each subject enrolled, a CRF will be completed and signed by the investigator or an authorized co-investigator. All forms will be filled out using a black indelible pen, and must be legible. Any errors in recording of data in the CRFs should be crossed through with a single line. Amended information should be entered next to the original entry, and any amendments should be initialed and dated. The original entry should never be obscured. Correction fluid should never be used.

Any changes to the CRF that occur after the investigator or authorized co-investigator has signed will require the investigator or authorized co-investigator to review and re-sign the CRF.

When changes to CRF data are necessary following removal of the original CRF from the study site, any such changes will be documented on data clarification/resolution forms, which will be submitted to the investigator for signature.

13.2. Investigator File

The investigator is responsible for maintaining all records to enable the conduct of the study to be fully documented. Copies of the protocol, study approval letters, all original informed consent forms, site copies of all CRFs, product dispensing and accountability logs, and all correspondence pertaining to the study should be kept by the investigator for 2 years after either the last approval of a marketing application or the formal discontinuation of the clinical development of the investigational product.

The investigator is responsible for maintaining a confidential subject identification code, which provides the unique link between named source records and anonymous CRF data for the sponsor. The investigator must arrange for the retention of this confidential list for 2 years (may be kept in a storage facility) after the completion or discontinuation of the study.

No study document should be destroyed without prior written agreement between the investigator and the sponsor. Should the investigator elect to assign the study documents to another party, or move them to another location, the sponsor must be notified.

13.3. Provision of Additional Information

On request, the investigator will supply the sponsor with additional data relating to the study, or copies of relevant source records. In case of particular issues or governmental queries, it is also necessary to have access to the complete study records, provided that the subject's confidentiality is protected in accordance with applicable regulations.

14. CHANGES IN THE CONDUCT OF THE STUDY

14.1. Protocol Amendments

Regulatory and IRB/IEC approval must be obtained (as per country requirements) for any change to this protocol that could affect the safety of the subjects, the scope/design of the study, any increase in dose of or duration of exposure to GAMMAPLEX, an increase in the number of subjects treated, the addition of a new test or procedure, or the dropping of a test intended to monitor safety.

14.2. Premature Study Termination

The sponsor reserves the right to terminate the study at any time. If the study is terminated, the CRO, the sponsor and the investigator will ensure that adequate consideration is given to the protection of the subjects' interests.

15. REPORTING AND PUBLICATION

15.1. Study Reports

15.1.1. Interim Clinical Study Report

A complete study report, in accordance with ICH guidelines, may be prepared by the CRO after the 30th evaluable adult subject has completed Day 32. All other subjects recruited up to this timepoint, e.g. non-evaluable subjects or children (applicable to subjects enrolled before implementation of Protocol Version 7) will be analyzed for safety, and those who have completed Day 32 will also be included. This report may be used for submission to the U.K. and EU regulatory authorities.

15.1.2. Final Clinical Study Report

After all subjects have completed the study, the CRO will prepare a final clinical study report in accordance with ICH guidelines. The sponsor will be responsible for filing Marketing Authorisation Applications (MAA) to countries other than the U.S. A copy of the final study report will be provided to all applicable regulatory authorities as required.

15.2. Publication Policy

The investigator and the CRO will discuss with the sponsor the possible preparation of a manuscript for publication in a peer-reviewed professional journal or an abstract for presentation, oral or written, to a learned society or symposium. Any of the parties may undertake the task but all must agree to the strategy before the work is started.

The investigator, the CRO, or their employees shall not be permitted to present at a meeting or to publish any results of the study without informing the sponsor. The sponsor shall have 60 days after receipt of a proposed publication to object to such proposed publication on reasonable grounds. For the avoidance of doubt the grounds that the subject matter is patentable or commercially sensitive shall constitute reasonable grounds.

Authorship should reflect work done by the investigators and personnel of the CRO and the sponsor, in accordance with generally recognized principles of scientific collaboration.

In accordance with standard editorial and ethical practice, the sponsor will support publication of multi-center trials only in their entirety and not as individual center data. A publications subcommittee will be established after recruitment is completed.

16. REFERENCES

1. Cines D, Blanchette S. Immune Thrombocytopenic Purpura. N Engl J Med 2002; 346: 995-1008.
2. George JN, Woolf SH, Raskob GE, et al. Idiopathic thrombocytopenic purpura: a practice guideline developed by explicit methods for the American Society of Hematology. Blood. 1996; 88:3-40.
3. British Committee for Standards in Haematology General Haematology Task Force Guidelines for the investigation and management of idiopathic thrombocytopenic purpura in adults, children and in pregnancy. British Journal of Haematology. 2003 120:574-96.
4. Buchanan GR, de Alarcon PA, Feig SA, et al. Acute idiopathic thrombocytopenic purpura –management in childhood. Blood. 1997; 89:1464-5.
5. Zimmer J, Andres E, Noel E, et al. Current management of adult idiopathic thrombocytopenic purpura in practice: a cohort study of 201 patients from a single center. Clin Lab Haematol. 2004; 26:137-42.
6. Portielje JE, Westendorp RG, Kluin-Nelemans HC, et al. Morbidity and mortality in adults with idiopathic thrombocytopenic purpura. Blood 2001; 97:2549-54.
7. Woerner SJ, Abildgaard CF, French BN. Intracranial hemorrhage in children with idiopathic thrombocytopenic purpura. Pediatrics 1981; 67:453-60.
8. Bolton-Maggs PH. Acute idiopathic thrombocytopenic purpura—management in childhood. Blood. 1997; 89:1465.
9. Imbach P, Barandun S, d'Apuzzo V, et al. High-dose intravenous gammaglobulin for idiopathic thrombocytopenic purpura in childhood. Lancet 1981; 1:1228-31.
10. Bussel JB, Kimberly RP, Inman RD, et al. Intravenous gammaglobulin treatment of chronic idiopathic thrombocytopenic purpura. Blood 1983; 62:480-6.
11. Bussel JB, Pham LC. Intravenous treatment with gammaglobulin in adults with immune thrombocytopenic purpura: review of the literature. Vox Sang. 1987; 52:206-11.
12. Carroll RR, Noyes WD, Rosse WF, et al. Intravenous immunoglobulin administration in the treatment of severe chronic immune thrombocytopenic purpura. Am J Med. 1984; 76:181-6.
13. Oral A, Nusbacher J, Hill JB, et al. Intravenous gammaglobulin in the treatment of chronic idiopathic thrombocytopenic purpura in adults. Am J Med. 1984; 76:187-92.

14. Warrier I, Lusher JM. Intravenous gammaglobulin treatment for chronic idiopathic thrombocytopenic purpura in children. Am J Med. 1984; 76:193-8.

15. Bussel JB, Eldor A, Kelton JG, et al. IGIV-C, a novel intravenous immunoglobulin: evaluation of safety, efficacy, mechanisms of action, and impact on quality of life. Throm Haemost 2004; 91:771-8.

16. Bussel JB, Schulman I, Hilgartner MW, et al. Intravenous use of gammaglobulin in the treatment of chronic immune thrombocytopenic purpura as a means to defer splenectomy. J Pediatr. 1983; 103:651-4.

17. Kurlander R, Coleman RE, Moore J, et al. Comparison of the efficacy of a two-day and a five-day schedule for infusing intravenous gammaglobulin in the treatment of immune thrombocytopenic purpura in adults. Am J Med. 1987; 83:17-24.

18. Mankarious S, Lee M, Fischer S, Pyun KH, Ochs HD, Oxelius VA, et al. The half-lives of IgG subclasses and specific antibodies in patients with primary immunodeficiency who are receiving intravenously administered immunoglobulin. J Lab Clin Med 1988; 112(5): 634-640.

19. Bio Products Laboratory. GAMMAPLEX® Human Normal Immunoglobulin, Intravenous. Investigator's Brochure – Idiopathic Thrombocytopenic Purpura. Edition 5, July 2009.

20. Parexel Clinical Pharmacology Research Unit. A comparison of the pharmacokinetics, safety and tolerance of two formulations of a liquid IVIG Vigam® Liquid and GAMMAPLEX® using standard and accelerated infusion rates in healthy adult volunteers (three treatment arms). 23 May 2005.

21. Gammagard® S/D Package Insert. Baxter Healthcare Corporation, Westlake Village, CA, Revised (April) 2005.

22. EMEA Committee for Proprietary Medicinal Products. Note for Guidance on the Clinical Investigation of Human Normal Immunoglobulin for Intravenous Administration (IVIG). London: 29 June 2000, CPMP/BPWG/388.95 rev. 1.

APPENDIX 1: ASSESSMENT BLEEDING/HEMORRHAGE

		Grade				
**Adverse Event**	**Short Name**	**1**	**2**	**3**	**4**	**5**
		**HEMORRHAGE/BLEEDING**				
Petechiae/purpura (hemorrhage/bleeding into skin or mucosa)	Petechiae	Few petechiae	Moderate petechiae; purpura	Generalized petechiae or purpura	-	-
Hematoma	Hematoma	Minimal symptoms, invasive intervention not indicated	Minimally invasive evacuation or aspiration indicated	Transfusion, interventional radiology, or operative intervention indicated	Life-threatening consequences; major urgent intervention indicated	Death
Hemorrhage/bleeding associated with surgery, intra-operative or postoperative	Hemorrhage with surgery	-	-	Requiring transfusion of 2 units non-autologous (10 cc/kg for pediatrics) pRBCs beyond protocol specification; postoperative interventional radiology, endoscopic, or operative intervention indicated	Life-threatening consequences	Death
Hemorrhage, CNS	CNS hemorrhage	Asymptomatic, radiographic findings only	Medical intervention indicated	Ventriculostomy, ICP monitoring, intraventricular thrombolysis, or operative intervention indicated	Life-threatening consequences; neurologic deficit or disability	Death
Hemorrhage, GI<br>– *Select*:<br>– Abdomen NOS<br>– Anus<br>– Biliary tree<br>– Cecum/appendix<br>– Colon<br>– Duodenum<br>– Esophagus<br>– Ileum<br>– Jejunum<br>– Liver<br>– Lower GI NOS<br>– Oral cavity<br>– Pancreas<br>– Peritoneal cavity<br>– Rectum<br>– Stoma<br>– Stomach<br>– Upper GI NOS<br>– Varices (esophageal)<br>– Varices (rectal)	Hemorrhage, GI – *Select*	Mild, intervention (other than iron supplements) not indicated	Symptomatic and medical intervention or minor cauterization indicated	Transfusion, interventional radiology, endoscopic, or operative intervention indicated; radiation therapy (i.e., hemostasis of bleeding site)	Life-threatening consequences; major urgent intervention indicated	Death
Hemorrhage, GU<br>– *Select*:<br>– Bladder<br>– Fallopian tube<br>– Kidney<br>– Ovary<br>– Prostate<br>– Retroperitoneum<br>– Spermatic cord<br>– Stoma<br>– Testes<br>– Ureter<br>– Urethra<br>– Urinary NOS<br>– Uterus<br>– Vagina<br>– Vas deferens	Hemorrhage, GU – *Select*	Minimal or microscopic bleeding; intervention not indicated	Gross bleeding, medical intervention, or urinary tract irrigation indicated	Transfusion, interventional radiology, endoscopic, or operative intervention indicated; radiation therapy (i.e., hemostasis of bleeding site)	Life-threatening consequences; major urgent intervention indicated	Death

		Grade				
**Adverse Event**	**Short Name**	**1**	**2**	**3**	**4**	**5**
		**HEMORRHAGE/BLEEDING**				
Hemorrhage, pulmonary/ upper respiratory<br>– *Select*:<br>– Bronchopulmonary NOS<br>– Bronchus<br>– Larynx<br>– Lung<br>– Mediastinum<br>– Nose<br>– Pharynx<br>– Pleura<br>– Respiratory tract NOS	Hemorrhage pulmonary – *Select*	Mild, intervention not indicated	Symptomatic and medical intervention indicated	Transfusion, interventional radiology, endoscopic, or operative intervention indicated; radiation therapy (i.e., hemostasis of bleeding site)	Life-threatening consequences; major urgent intervention indicated	Death
Hemorrhage/Bleeding – other (specify, __)	Hemorrhage – other (specify)	Mild without transfusion	–	Transfusion indicated	Catastrophic bleeding, requiring major nonelective intervention	Death

Excerpted from Common Terminology Criteria for Adverse Events v3.0 (CTCAE). 12 December, 2003, pp 30–33.

APPENDIX 2: INVESTIGATOR AGREEMENT

INVESTIGATOR AGREEMENT

A PHASE III, MULTICENTER, OPEN-LABEL STUDY TO EVALUATE THE EFFICACY AND SAFETY OF GAMMAPLEX® IN CHRONIC IDIOPATHIC THROMBOCYTOPENIC PURPURA (Protocol Number GMX02)

I have been adequately informed about the development of the investigational product to date and agree that this study protocol contains all the information required to conduct the study.

I will provide copies of the study protocol and all product information relating to prior product experience furnished to me by the sponsor/CRO to all staff responsible to me who participate in this study. I will discuss this material with them to ensure that they are fully informed regarding the product and the conduct of the study.

The information contained in this document is CONFIDENTIAL and, except to the extent necessary to obtain informed consent, may not be disclosed, unless such disclosure is required by government regulation or state/local customs or law. Persons to whom the information is disclosed must be informed that the information is CONFIDENTIAL and cannot be disclosed by them.

By my signature below I agree to conduct this clinical trial in accordance with the protocol, Good Clinical Practice, the Declaration of Helsinki, government regulations, and state/local customs or laws, including those applying to institutional/ethics review and informed consent.

I agree to ensure the confidentiality of the subjects enrolled under my care; however, I agree to make their medical records available to authorized representatives of INC Research, Inc. (U.S.), GVKBIO (India) and Activa-Cro (Argentina); authorized representatives of Bio Products Laboratories, the sponsor of this clinical trial; and relevant regulatory authorities.

I have read the Investigator's Brochure and protocol, and I am aware of my responsibilities as investigator as stated on FDA Form 1572.

Site Investigator:

Investigator name: ______________________ Site Name: ______________________

Signature: ______________________ Date: ______________________
